# Low-temperature preparation of enzyme-treated sesame paste: Quality enhancement and hazard mitigation

**DOI:** 10.1016/j.fochx.2026.104341

**Published:** 2026-08-29

**Authors:** Lei Jin, Yu-Xiang Ma, Li-Xia Hou, Xue-De Wang, Jin-chu Yang, Yong-ming Xu, Hua-Min Liu

**Affiliations:** a College of Food Science and Technology & Institute of Special Oilseed Processing and Technology, Henan University of Technology, Zhengzhou 450001, China; b Technology Center, China Tobacco Henan Industrial Co., Ltd., Zhengzhou 450000, China

**Keywords:** Enzymatic treatments, Sesame paste, Volatile compounds, Heterocyclic amine, Rheological properties

## Abstract

This research evaluated the impact of combined enzymatic treatments on the physicochemical properties of sesame paste. The quality attributes of sesame paste were systematically characterized, including texture properties, heterocyclic amine content, stability, volatile compounds, and microstructural characteristics. The results showed that the amino acid and polysaccharide content of the samples decreased significantly after the combined enzymatic treatments. Concurrently, the total color difference and volatile compound content increased with the extent of the Maillard reaction. Notably, enzymatic pretreatment with a cellulase–papain complex promoted thermal reactions at lower temperatures, producing a paste similar in color, flavor, and stability to that produced at higher temperatures, while reducing the formation of heterocyclic aromatic amines. These results indicate that stable, high-quality sesame paste can be produced by promoting the Maillard reaction through enzymatic treatment. This strategy provides new insights and methods for the industrial production of premium sesame paste products.

## Highlights

• Enzymatic treatment released Maillard precursors, enhancing sesame paste quality.
• Cellulase-papain complex boosted volatiles and color at lower roasting temperature.
• Enzyme treatment reduced particle size and significantly minimized oil separation.
• Lower-temperature enzymatic processing successfully mitigated hazardous HAs.
• This strategy achieved high temperature paste qualities while ensuring food safety.

## Graphical abstract

**Figure:**
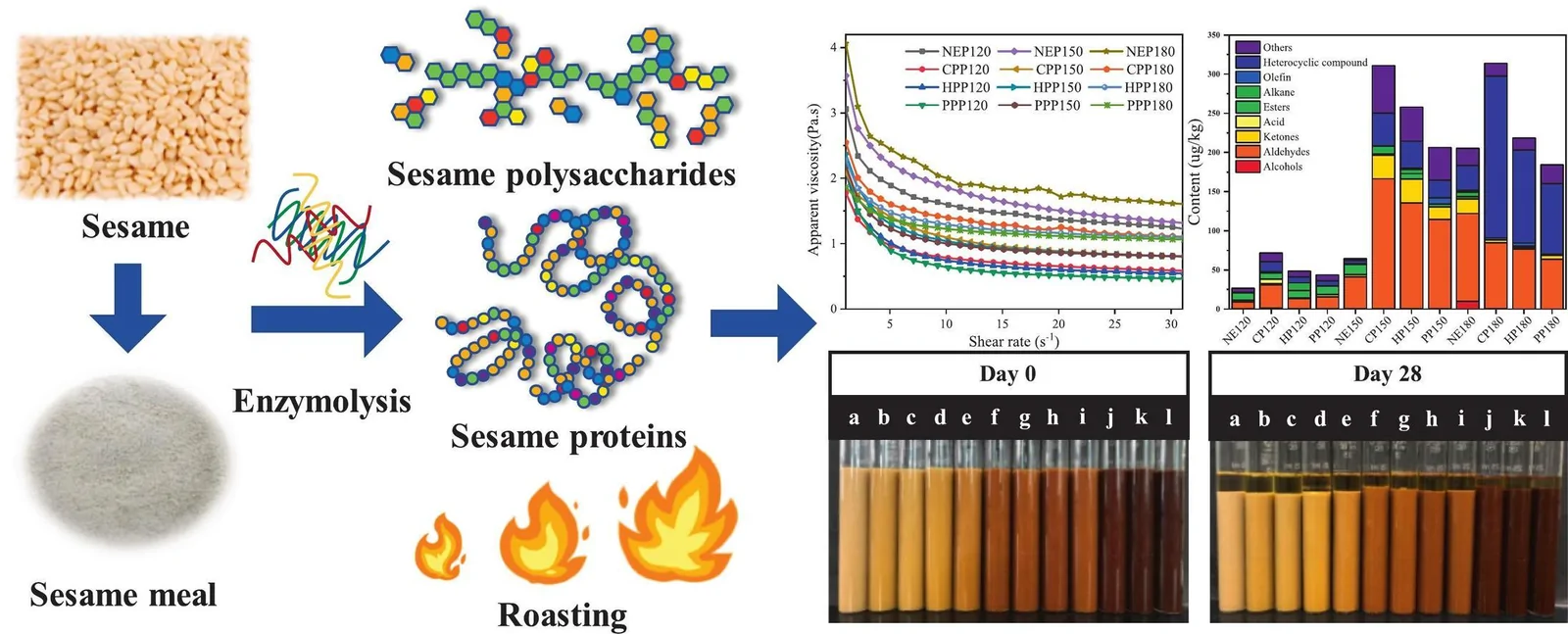

## 1 Introduction

Sesame paste provides high nutritional value, serving as an excellent source of proteins, minerals, unsaturated fatty acids, and dietary fiber (Jin, Guo, et al., 2022). This product is extensively consumed globally, particularly in Asia, Africa, Europe, and South America (Yang et al., 2024). However, sesame paste is a highly concentrated colloidal suspension in which protein-rich, hydrophilic solid particles are dispersed within a continuous oil phase. During storage, the system is prone to phase separation, leading to quality instability and lipid oxidation (Guo et al., 2024). Consequently, this phenomenon severely compromises consumer acceptance and restricts the commercial development of high-quality sesame paste products.

To produce sesame paste with stable quality and a rich flavor profile, sesame seeds are typically subjected to roasting (He et al., 2021). During this process, the Maillard reaction serves as the core reaction, driving the condensation between the carbonyl groups of reducing carbohydrates and the amino groups of proteins (Liu et al., 2020), which generates diverse volatile flavor compounds and imparts an appealing characteristic flavor to the sesame paste. Simultaneously, both the textural properties and the overall stability of the food system can be enhanced by the resulting carbohydrate-protein conjugates (Starowicz & Zieliński, 2019). Nevertheless, intense thermal treatment can lead to quality deterioration and induce the formation of harmful substances, such as heterocyclic aromatic amines (HAAs), thereby raising safety concerns (Yang et al., 2024). Consequently, it is imperative to develop innovative strategies that simultaneously maintain product quality and reduce the formation of heat-induced toxicants.

Recently, enzymatic treatment has attracted considerable interest in the fields of food quality enhancement and flavor development, largely due to its mild processing conditions, high efficiency, and excellent controllability. Enzymatic hydrolysis effectively degrades macromolecules in sesame, such as polysaccharides and proteins, into reducing sugars and free amino acids. As crucial precursors, these small molecules can facilitate the Maillard reaction at relatively low temperatures. Xu et al. (2025) reported that sunflower seed oil with a desirable flavor profile was prepared at low temperatures using Viscozyme L and Alcalase. Sesame meal, the principal by-product of sesame oil production, contains abundant proteins (30–50%) and carbohydrates (15–28%), serving as a high-quality substrate for the Maillard reaction (Eze et al., 2025). Currently, sesame meal is predominantly utilized as low-value-added animal feed, and its potential has not been fully realized. While previous studies have explored the enzymatic hydrolysis of sesame meal to boost food flavor and modify the physicochemical properties of foods (Hu et al., 2021; Hu et al., 2025), an investigation into the application of enzyme-treated sesame meal for preparing high-quality and low-hazard sesame paste remains lacking.

Therefore, in this study, three complex enzymes were employed for the enzymatic hydrolysis of sesame meal, and the resulting hydrolysates were applied as precursors in the preparation of sesame paste. The relationship between various enzymatic treatments of sesame meal and the quality of sesame paste was elucidated through a comprehensive analysis of amino acid profiles, volatile compounds, HAAs, texture, particle size distribution, rheological properties, oil separation rates, and microstructural characteristics. These findings are expected to provide valuable insights for the commercial manufacturing of high-quality sesame paste.

## 2 Materials and methods

### 2.1 Materials

The white sesame seeds utilized in this study were obtained from the Henan Academy of Agricultural Sciences (Henan, China). Prior to experimental analysis, all samples were preserved at −20 °C in airtight packaging. The four enzymes used in the study–namely, cellulase (activity of 400 U/mg; CAS: 9012-54-8), hemicellulase (activity of 20 U/mg; CAS: 9025-56-3), pectinase (activity of 50 U/mg; CAS: 9032-75-1), and papain (activity of 800 U/mg; CAS: 9001-73-4) – were procured from Shanghai Yuanye Bio-Technology Co., Ltd. Oil was cold-pressed from some of the sesame kernels by a yz-150 hydraulic press (Hengan, China).

### 2.2 Preparation of defatted seed meal

Following sesame kernel extraction by dry decortication, the samples were ground using an FW-100 crusher (Jintan, China). Then the ground sesame kernels were defatted with petroleum ether (boiling range: 30–60 °C) for 7 h at 45 °C. Finally, the defatted seed meal obtained by the above steps was blended with 80% ethanol for 4 h to eliminate free sugars and free amino acids. The above solids were dried for later use.

### 2.3 Enzymatic treatment

Defatted seed meal (100 g) and citric acid buffer solution (490 mL; pH 5.0) were poured into each of four glass flasks (800 mL). Subsequently, measured amounts of the cellulase-papain, hemicellulase-papain, and pectinase-papain complexes were individually dissolved in 10 mL of citrate buffer and added to separate glass flasks. One flask contained only 100 g seed meal and 500 mL citric acid buffer solution without enzymes as a control. These materials were mixed thoroughly and shaken for 3 h in an incubator at 50 ± 1 °C (STC—C, XinMiao, China). The glass flasks were then heated at 97 °C for 10 min (DZKW-S-4, YongGuangMing, China) to deactivate the enzymes. Finally, the three enzymatically treated seed meals and control were freeze-dried (Wu et al., 2010). The three samples enzymatically treated by cellulase-papain complex, hemicellulase-papain complex, and pectinase-papain complex were designated as CP, HP, and PP, respectively. Seed meal without enzyme treatment was designated as NE.

### 2.4 Roasting and grinding

Each seed meal sample (100 g) was placed in an open roaster and heated in a DF-101 oil bath (YuHua, China) for 30 min under continuous mechanical stirring to ensure uniform heating. The samples roasted at 120 °C, 150 °C and 180 °C were designated as NE120, NE150, NE180, CP120, CP150, CP180, HP120, HP150, HP180, PP120, PP150, and PP180.

Then, samples of each of the 9 enzymatically-treated defatted sesame meals and 3 controls were mixed with cold-pressed sesame oil in a 1:1 ratio (*w*/w), producing sesame paste. These paste samples were designated as NEP120, NEP150, NEP180, CPP120, CPP150, CPP180, HPP120, HPP150, HPP180, PPP120, PPP150, and PPP180.

### 2.5 Meal samples

#### 2.5.1 Proximate composition

Moisture, fat, protein, and polysaccharides in the roasted samples (i.e., NE120, NE150, NE180, CP120, CP150, CP180, HP120, HP150, HP180, PP120, PP150, and PP180) were determined by previous analytical methods with a slight modification (Jin, Guo, et al., 2022). To determine the water content, the specimens were dehydrated in a drying oven (Model DHG-9140A, Jinghong, China) at 106 °C for 80 min. To evaluate fat content, samples were initially subjected to Soxhlet extraction, and the obtained fat were subsequently oven-dried to a constant mass. The polysaccharide content was determined by measuring the absorbance of phenol/sulphuric acid reaction liquid at 490 nm (UV-2401PC, Shimadzu, Japan). Protein content was calculated by digestion at 420 °C for 40 min and titration with hydrochloric acid.

#### 2.5.2 Amino acid composition

Each sample (20 mg) was put into a vial, and 6 M HCl was added for 24 h at 110 °C to hydrolyze samples. Following hydrolysis, the reaction liquid was diluted, and amino acid composition was determined by a JSYK0-S-433D auto-analyzer (Sykam, Germany) equipped with a Zorbax 80A C_18_ column (Ruan et al., 2025).

#### 2.5.3 Solid-phase extraction of heterocyclic aromatic amines

Norharman and harman are the major heterocyclic aromatic amines formed during the roasting of oilseeds (Liu et al., 2022). The extraction and purification of norharman and harman were performed according to the method described by Zhang, Wang, et al. (2023), with some modifications. In brief, 1 g of the sesame meal and 5 mL acetonitrile were added into a centrifuge tube, subjected to ultrasound for 15 min and centrifuged at 4500*g* for 4 min. Subsequently, the supernatant containing norharman and harman was loaded onto solid-phase extraction cartridges, which were then successively rinsed with 0.1 M HCl and pure methanol.

#### 2.5.4 Heterocyclic aromatic amines measurement

The concentrations of norharman and harman in the samples were determined using a Waters Alliance 2695 HPLC system coupled with a Waters 2475 fluorescence detector (Waters, Milford, MA, USA). The excitation and emission wavelengths were set at 300 and 440 nm, respectively. The chromatographic column was Waters SunFire C_18_. The chromatographic conditions were as follows: column temperature, 30 °C; injection volume, 10 μL; mobile phase A, 10 mM ammonium formate–formic acid solution (pH 3.0); mobile phases, 85% 10 mM ammonium formate-formic acid solution (pH = 3) for A and 15% acetonitrile for B; and flow rate, 0.8 mL/min (Zhang, Li, et al., 2023). For norharman, the LOD, LOQ, recovery, and RSD were 0.05 ng/mL, 0.22 ng/mL, 88.69%, and 5.52%, respectively. For harman, the corresponding values were 0.03 ng/mL, 0.10 ng/mL, 81.89%, and 13.61%, respectively.

#### 2.5.5 Extraction of volatile compounds by headspace solid-phase microextraction (HS-SPME)

HS-SPME was conducted in accordance with the method of Jin, Zhao, et al. (2022). 4 g of sesame meal (NE120, NE150, NE180, CP120, CP150, CP180, HP120, HP150, HP180, PP120, PP150, and PP180) and 10 μL of 4-nonanol were added to an extraction vial (20 mL). Polydimethylsiloxane/divinylbenzene/carboxen (PDMS/DVB/CAR) fibers (film thickness: 50/30 μm) were used in this experiment. Before each extraction, a fiber was thermally conditioned at 250 °C for 60 min and then exposed to a sample for 40 min.

#### 2.5.6 Gas chromatography-mass spectrometry (GC–MS) analysis

The volatile compounds were identified by a QP2010 GC–MS with a Rtx-5MS chromatographic column (Shimadzu, Japan) according to the reported method (Yin et al., 2021). The protocol was as follows: an initial temperature of 40 °C, increased to 220 °C at a rate of 6 °C/min, and finally ramped to 250 °C at 15 °C/min. The ion source and port temperatures were 230 °C and 250 °C, respectively. Initial identification of volatile components was performed through mass spectral comparison with the NIST 17 library.

### 2.6 Paste samples

#### 2.6.1 Color measurement

The color measurements of the paste samples (NEP120, NEP150, NEP180, CPP120, CPP150, CPP180, HPP120, HPP150, HPP180, PPP120, PPP150, and PPP180) were performed on a CR-410 chromameter based on the methods of Gul et al. (2024). Instrument was standardized utilizing a white calibration tile before color measurements.

#### 2.6.2 Particle size distribution

Particle size distribution was determined based on a previous method (Miao et al., 2022). Briefly, 50 mL water and approximately 2 g of one of the 12 paste samples were placed in a centrifuge tube, and then vortexed (MX-S, DLAB, China). Particle-size distribution was measured by a Mastersizer 3000 analyzer (Malvern, Worcestershire, UK). The refractive indexes of sesame paste and water are 1.475 and 1.333, respectively.

#### 2.6.3 Stability analysis

15 g of each prepared paste was transferred into transparent vials, which were stored at 25 °C in ambient light, and photographed on days 0, 4, 14, and 28 of storage.

15 g of each prepared paste was transferred into plastic tubes and centrifuged at 4000 ×*g* for 5 min (TD5B, Henan, China). The oil was removed from the tube and weighed. The degree of oil separation was determined utilizing the following formula:

(1) $$\eta \;\left(g/100\;g\right)=\frac{m_{0}}{15}\times 100$$

where *m*_o_ and *η* denote the mass of the extracted oil and the centrifugal oil separation rate, respectively.

#### 2.6.4 Rheological measurements

The rheological data of the twelve samples were obtained using a rheometer (HAAKE MARS, Germany) at room temperature. Sesame paste samples were stirred, left for 20 min and then placed on a parallel plate (40 mm diameter). The rheological behavior of the samples was characterized utilizing the power-law equation, expressed as (Jin, Guo, et al., 2022):

(2) $$\tau =K{\dot{\gamma }}^{n}$$

where $\dot{\gamma }$, *τ*, *K*, and *n* indicate shear rate (s^−1^), shear stress (Pa), consistency coefficient (Pa·s^n^), and flow behavior index, respectively.

In order to plot the storage and loss moduli curves, frequency sweep was determined at 1% strain over a range of 0.1–10 rad/s.

#### 2.6.5 Texture

Following the protocol established by Gul et al. (2024), the textural characteristics of the samples were determined. This assessment was conducted utilizing a TA-XT instrument (Stable Micro System, England) fitted with a P25 probe. The 12 paste samples were carefully placed into 50 mL containers. The speed of P25 probe was 2.00 mm/s during test, and the trigger force was 10 g.

#### 2.6.6 Microstructure imaging

The 12 paste samples were stained with FITC (25 μL) and Nile red (25 μL) to reveal protein and lipid, respectively (Yuan et al., 2026). The microstructure was visualized using a confocal laser scanning microscope (LSM710 Carl Zeiss AG, Germany), which incorporated dual laser excitation (Ar/Kr at 488 nm and He/Ne at 633 nm).

### 2.7 Statistical analysis

Triplicate independent trials were carried out for all experiments, and the results are depicted as mean ± SD. For statistical evaluation, the data were processed via the SPSS (version 21.0). Statistical significance was predefined at *P* < 0.05. Furthermore, Origin 2023 (OriginLab Corp., USA) was utilized to construct all graphical illustrations.

## 3 Results and discussion

### 3.1 Proximate composition and HAAs content of the sesame meal

The proximate components of the 12 meal samples are presented in Table S1. At the same roasting temperature, the protein and crude polysaccharide contents differed significantly between enzyme-treated and non-enzyme-treated samples; furthermore, protein and crude polysaccharide contents in all four treatment groups decreased significantly as roasting temperature increased (*P* < 0.05). Among the 12 samples, CP180 showed the lowest contents of protein and crude polysaccharide. This indicated that the cellulase-papain complex efficiently degraded macromolecular proteins and polysaccharides into low-molecular-weight compounds. Subsequently, at the high-temperature condition of 180 °C, these low-molecular-weight products acted as thermal reactive precursors and were extensively consumed via the Maillard reaction, thereby substantially reducing their contents (Mohamed Ahmed et al., 2021). Meanwhile, the side chain sugar units of polysaccharides, which provided precursors for the Maillard reaction, were more easily degraded at high roasting temperatures, thereby reducing crude polysaccharide content (Liu et al., 2020). In addition, the fat content rose with the increase of roasting temperature, such that CP180 had the highest fat content (2.50 g/100 g) among all samples. This can be explained by the fact that the losses of protein, moisture, and crude polysaccharide relatively elevated the fat content. Similarly, Harivaindaran et al. (2023) reported an increase in the measured fat content of roasted black seeds, which was attributed to moisture loss and improved lipid extractability.

The ingestion of HAAs is widely acknowledged to jeopardize human health owing to their potent mutagenicity. Unlike the aminoimidazoazaarene HAAs commonly associated with cooked meat, norharman and harman belong to the β-carboline subclass and have been reported in roasted oilseeds and oilseed-derived products (Liu et al., 2022; Pfau & Skog, 2004; Zhang et al., 2020). Therefore, we conducted a quantitative analysis of the levels of norharman and harman in samples of roasted sesame meal. As shown in Table S1 and Fig. S3, the contents of norharman and harman exhibited similar trends. At 120 °C and 150 °C, there was no noticeable difference between the enzyme-treated and non-enzyme-treated samples (*P* > 0.05), and eight samples had low levels of norharman (0.45 ng/mL – 4.44 ng/mL) and harman (0.02 ng/mL – 0.95 ng/mL), indicating that the HAAs of sesame meal could be kept low by roasting at lower temperatures. However, the norharman and harman contents of sesame meal roasted at 180 °C increased rapidly, such that the HAAs of CP180, HP180, and PP180 were significantly higher than those of NE180 (*P* < 0.05). The observed elevations can be ascribed to the prior enzymatic hydrolysis, which effectively cleaved macromolecules—specifically proteins and polysaccharides—into reactive precursors such as free amino acids and reducing sugars. At 180 °C, these precursors rapidly promoted the Maillard reaction and subsequent Pictet-Spengler condensations, ultimately leading to the accelerated formation of HAAs (Gibis, 2016).

### 3.2 Amino acid composition analysis of sesame meal

As shown in Table 1, both enzyme treatment and roasting temperature significantly affected the total amino acid content of the samples (*P* < 0.05). NE120 exhibited the highest total amino acid content, at 45.72 mg/100 mg, whereas CP180 showed the lowest value, at 39.01 mg/100 mg. Within each enzyme treatment, the total amino acid content decreased significantly as the roasting temperature increased from 120 to 180 °C (*P* < 0.05). At the same roasting temperature, the CP and HP groups had significantly lower total amino acid contents than the corresponding NE groups (*P* < 0.05), indicating that the effects of enzyme treatment on total amino acid content depended on the enzyme combination used. The contents of most individual amino acids also tended to decrease with increasing roasting temperature, with lysine showing a particularly pronounced reduction. Compared with NE120, the lysine contents of CP180 and HP180 decreased by 36.9% and 41.8%, respectively, suggesting that lysine loss was associated with enzymatic pretreatment and high-temperature roasting. The decrease in lysine content may be related to its participation in Maillard-related and other heat-induced reactions. Inan-Eroglu et al. (2020) reported that the highly reactive ε-amino group in the lysine side chain makes lysine particularly susceptible to glycoxidation, which is consistent with the significant decrease in lysine content observed in the present study.

**Table 1:** Amino acid composition of sesame meal with different treatments.

Amino acid (mg/100 mg)	Samples of sesame meal with different treatments⁎											
	NE120	CP120	HP120	PP120	NE150	CP150	HP150	PP150	NE180	CP180	HP180	PP180
Asp	3.91 ± 0.08^Aa^	3.81 ± 0.06^Aa^	3.80 ± 0.04^Aa^	3.86 ± 0.05^Aa^	3.87 ± 0.04^Aa^	3.70 ± 0.06^Ab^	3.85 ± 0.04^Aa^	3.75 ± 0.04^Bb^	3.73 ± 0.05^Ba^	3.76 ± 0.05^Aa^	3.51 ± 0.03^Bb^	3.78 ± 0.04^ABa^
Thr	1.74 ± 0.02^Aa^	1.68 ± 0.03^Ab^	1.68 ± 0.01^Ab^	1.69 ± 0.02^Ab^	1.62 ± 0.03^Bb^	1.45 ± 0.02^Bd^	1.49 ± 0.01^Cc^	1.67 ± 0.02^Aa^	1.62 ± 0.02^Ba^	1.34 ± 0.03^Cc^	1.51 ± 0.01^Bb^	1.61 ± 0.02^Ba^
Ser	2.22 ± 0.03^Ba^	2.14 ± 0.02^Ab^	2.14 ± 0.03^Ab^	2.17 ± 0.02^Ab^	2.33 ± 0.02^Aa^	1.99 ± 0.01^Bd^	2.07 ± 0.01^Bc^	2.11 ± 0.03^Bb^	2.12 ± 0.02^Ca^	1.79 ± 0.02^Cd^	1.87 ± 0.03^Cc^	1.99 ± 0.01^Cb^
Glu	9.76 ± 0.05^Aa^	9.65 ± 0.08^Aa^	9.42 ± 0.07^Ab^	9.42 ± 0.04^Ab^	9.42 ± 0.05^Ba^	9.30 ± 0.06^Bb^	8.93 ± 0.07^Bc^	9.27 ± 0.05^Bb^	9.34 ± 0.06^Ba^	8.92 ± 0.04^Cd^	9.05 ± 0.08^Bc^	9.19 ± 0.05^Bb^
Gly	2.39 ± 0.02^Aa^	2.30 ± 0.02^Ab^	2.29 ± 0.02^Ab^	2.32 ± 0.03^Ab^	2.23 ± 0.02^Ba^	2.03 ± 0.01^Bc^	2.24 ± 0.02^Bb^	2.30 ± 0.03^Ab^	2.20 ± 0.02^Ba^	1.82 ± 0.03^Cc^	2.01 ± 0.02^Cb^	2.18 ± 0.02^Ba^
Ala	2.30 ± 0.01^Aa^	2.14 ± 0.02^Ac^	2.23 ± 0.01^Ab^	2.21 ± 0.02^Ab^	2.09 ± 0.02^Bb^	1.93 ± 0.01^Bd^	2.01 ± 0.03^Bc^	2.17 ± 0.02^Ba^	2.05 ± 0.02^Ca^	1.72 ± 0.02^Cd^	1.93 ± 0.02^Cc^	2.00 ± 0.01^Cb^
Cys	0.59 ± 0.02^Ab^	0.58 ± 0.01^Ab^	0.58 ± 0.01^Bb^	0.72 ± 0.02^Aa^	0.62 ± 0.02^Ab^	0.58 ± 0.01^Ac^	0.76 ± 0.01^Aa^	0.57 ± 0.01^Bc^	0.20 ± 0.01^Cc^	0.39 ± 0.01^Ba^	0.34 ± 0.01^Cb^	0.35 ± 0.01^Cb^
Val	2.19 ± 0.02^Aa^	2.08 ± 0.04^Ab^	2.19 ± 0.01^Aa^	2.22 ± 0.02^Aa^	2.05 ± 0.03^Bb^	2.02 ± 0.02^ABb^	2.06 ± 0.02^Bb^	2.11 ± 0.03^Ba^	2.08 ± 0.02^Ba^	1.98 ± 0.04^Bb^	1.97 ± 0.01^Cb^	1.97 ± 0.03^Cb^
Met	1.22 ± 0.01^Aa^	1.21 ± 0.01^Aa^	1.16 ± 0.02^Ab^	1.23 ± 0.03^Aa^	1.03 ± 0.01^Cc^	1.13 ± 0.02^Ba^	0.98 ± 0.01^Cd^	1.08 ± 0.02^Cb^	1.13 ± 0.02^Ba^	1.06 ± 0.01^Cb^	1.07 ± 0.03^Bb^	1.13 ± 0.01^Ba^
Ile	1.82 ± 0.01^Aa^	1.74 ± 0.03^Bb^	1.78 ± 0.02^Aa^	1.79 ± 0.02^Aa^	1.74 ± 0.03^Bbc^	1.79 ± 0.01^Aa^	1.71 ± 0.02^Bc^	1.76 ± 0.02^Aab^	1.67 ± 0.05^Cab^	1.65 ± 0.02^Cab^	1.59 ± 0.04^Cb^	1.71 ± 0.06^Aa^
Leu	3.25 ± 0.02^Aa^	3.08 ± 0.03^Ac^	3.16 ± 0.02^Ab^	3.21 ± 0.02^Aa^	3.11 ± 0.05^Ba^	2.92 ± 0.03^Bb^	3.04 ± 0.04^Ba^	3.11 ± 0.03^Ba^	3.02 ± 0.02^Ca^	2.72 ± 0.03^Cc^	2.71 ± 0.04^Cc^	2.90 ± 0.02^Cb^
Tyr	1.84 ± 0.02^Aa^	1.71 ± 0.03^Ab^	1.81 ± 0.03^Aa^	1.79 ± 0.03^Aa^	1.75 ± 0.02^Ba^	1.67 ± 0.03^Ab^	1.60 ± 0.03^Bc^	1.79 ± 0.02^Aa^	1.78 ± 0.03^Ba^	1.55 ± 0.02^Bd^	1.60 ± 0.01^Bc^	1.67 ± 0.03^Bb^
Phe	2.21 ± 0.02^Aa^	2.06 ± 0.02^Ac^	2.13 ± 0.03^Ab^	2.09 ± 0.02^Abc^	2.12 ± 0.02^Ba^	1.99 ± 0.03^Bb^	1.95 ± 0.02^Bb^	2.11 ± 0.02^Aa^	2.11 ± 0.03^Ba^	1.84 ± 0.02^Cd^	1.94 ± 0.02^Bc^	2.04 ± 0.03^Bb^
His	1.36 ± 0.02^Ba^	1.31 ± 0.01^Aa^	1.32 ± 0.02^Ba^	1.31 ± 0.03^Aa^	1.49 ± 0.02^Aa^	1.30 ± 0.03^Ab^	1.52 ± 0.02^Aa^	1.27 ± 0.01^Ab^	1.30 ± 0.02^Ca^	1.22 ± 0.02^Bb^	1.18 ± 0.03^Cb^	1.21 ± 0.02^Bb^
Lys	1.22 ± 0.02^Ab^	1.16 ± 0.02^Ac^	1.24 ± 0.02^Ab^	1.28 ± 0.01^Aa^	1.24 ± 0.02^Aa^	1.06 ± 0.01^Bb^	1.04 ± 0.01^Bb^	1.24 ± 0.02^Ba^	0.94 ± 0.01^Ba^	0.77 ± 0.02^Cc^	0.71 ± 0.02^Cd^	0.86 ± 0.02^Cb^
Arg	6.17 ± 0.02^Aa^	5.92 ± 0.06^Ac^	6.03 ± 0.02^Ab^	6.04 ± 0.03^Ab^	5.82 ± 0.04^Ba^	5.58 ± 0.03^Bb^	5.52 ± 0.05^Bb^	5.83 ± 0.03^Ba^	5.75 ± 0.04^Ca^	5.15 ± 0.03^Cc^	5.25 ± 0.06^Cb^	5.76 ± 0.02^Ca^
Pro	1.53 ± 0.03^Aa^	1.51 ± 0.02^Aa^	1.45 ± 0.03^Ab^	1.49 ± 0.01^Aab^	1.43 ± 0.02^Bab^	1.43 ± 0.02^Bab^	1.37 ± 0.02^Bb^	1.47 ± 0.04^Aa^	1.36 ± 0.03^Ca^	1.34 ± 0.02^Ca^	1.33 ± 0.03^Ba^	1.36 ± 0.02^Ba^
Total	45.72 ± 0.41^Aa^	44.06 ± 0.53^Ab^	44.42 ± 0.39^Ab^	44.83 ± 0.42^Ab^	43.96 ± 0.47^Ba^	41.83 ± 0.38^Bb^	42.13 ± 0.43^Bb^	43.60 ± 0.46^Ba^	42.40 ± 0.52^Ca^	39.01 ± 0.44^Cb^	39.57 ± 0.51^Cb^	41.72 ± 0.43^Ca^

⁎ NE represents sesame meal without enzyme treatment; CP, HP, and PP represent sesame meal enzymatically treated by cellulase-papain complex, hemicellulase-papain complex, and pectinase-papain complex, respectively. Numbers 120, 150, 180 correspond to the sesame meal roasted at 120 °C, 150 °C, and 180°C, respectively. Different uppercase letters (A–C) indicate significant differences among roasting temperatures within the same enzymatic treatment, whereas different lowercase letters (a–d) indicate significant differences among enzymatic treatments at the same roasting temperature (*P* < 0.05).

### 3.3 Volatile components and principal component analysis (PCA) of sesame meal

**Fig. 1:**
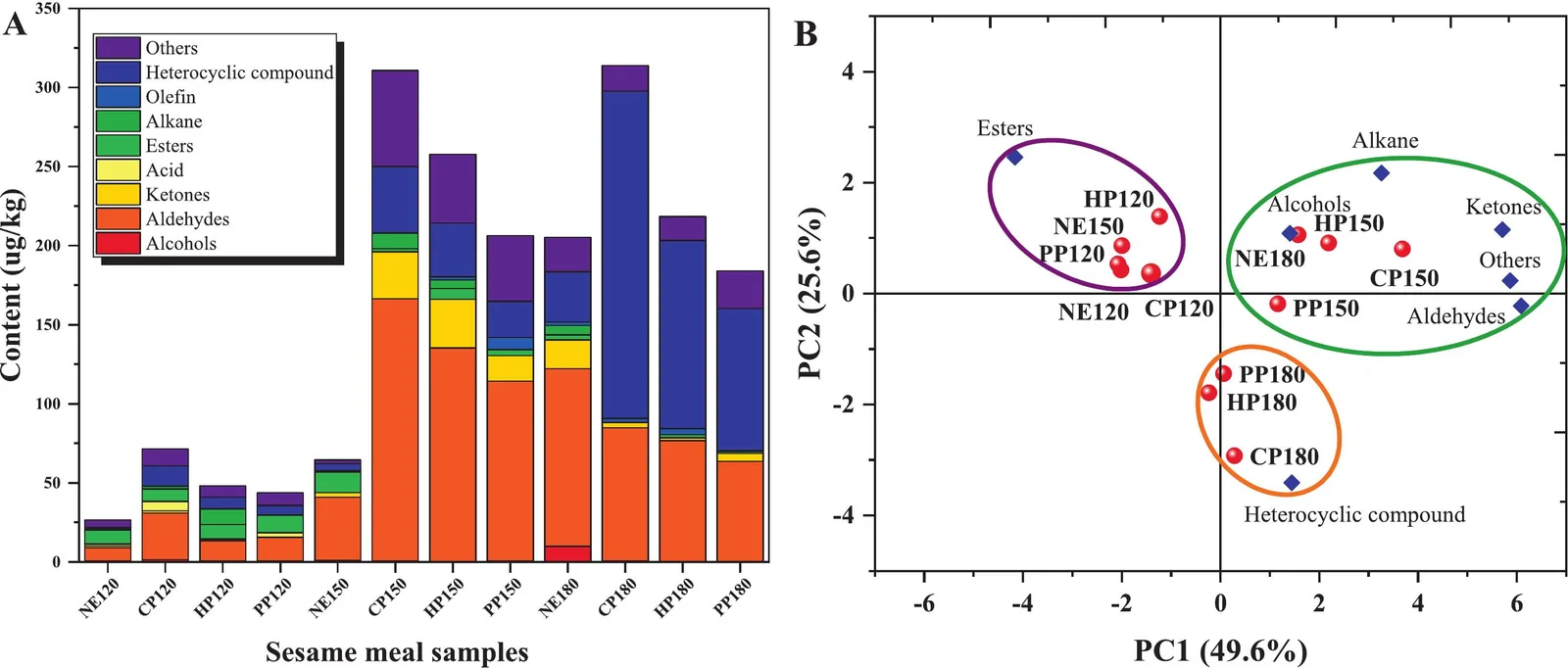
Volatile compounds (A) and principal component analysis (B) of twelve sesame meal samples. PC1 and PC2 represent the first and second principal components, respectively, accounting for 49.6% and 25.6% of the total variance.

A total of 163 volatile compounds were characterized in sesame meal (Table S2), and the heatmap revealed differences in their relative abundances among the samples (Figs. S1 and S4). These differences may be associated with the Strecker degradation of amino acids, in which amino acids act as reaction precursors and are converted into aldehydes and other small-molecule volatile compounds. Carbohydrate degradation and Maillard-related reactions involving carbohydrate- and protein-derived precursors may also contribute to the formation of these volatile compounds during roasting (Liu et al., 2020; Ma et al., 2022). As seen in Fig. 1A, volatile compounds detected in the sesame meal can be divided into alcohols, aldehydes, ketones, acids, esters, alkanes, olefin, and heterocyclic compounds. Aldehydes were among the major volatile compounds detected in all samples. During roasting, the thermal degradation of carbohydrates and proteins may generate low-molecular-weight carbonyl and amino compounds, which can subsequently participate in Maillard-related reactions and contribute to aldehyde formation. Guo et al. (2023) also reported that roasting induced structural changes and degradation of sesame polysaccharides and proteins, which may increase the availability of low-molecular-weight precursors involved in Maillard-related reactions and aldehyde formation. However, aldehydes, as seen in the figure, exhibited an initial increase followed by a subsequent decline as the roasting temperature increased. This pattern suggests that aldehydes acted as intermediate products in the Maillard reaction and were subsequently consumed in further reaction stages, resulting in a reduced aldehyde content (Guo et al., 2022). Meanwhile, the aldehydes content was higher in enzyme-treated samples, especially those treated with cellulase and papain, than non-enzyme-treated samples, because the treatment with cellulase and papain increased the content of free sugars and amino acids, increasing the reaction rate. For example, benzaldehyde and phenylacetaldehyde often have floral, malty, and toasted aromas. These compounds were produced from phenylalanine during the heating process, leading to a diminished concentration of phenylalanine (Zhao et al., 2017). This finding was similar to that of the amino acid analysis.

In addition to aldehydes, heterocyclic compounds were also major volatile compounds found in all samples, and all showed increases with rising temperature. As more small molecule sugars and peptides were created from the high-temperature degradation of polysaccharides and protein in the sesame meal, the Maillard reaction was promoted. This could be a reason why the content of heterocyclic compounds was different (Guo et al., 2023). For example, pyrans and furans, which typically give fruity, caramelized, and sweet notes, were formed via cyclization and 1,2-enolization of reduced sugar in the Maillard reaction (Li et al., 2020). Pyrazines, with cocoa-like, bread-like, and roasted aromas, were produced from the carbonyl compounds via the Maillard reaction. Differences in heterocyclic compounds between no-enzyme-treated and enzyme-treated samples were believed to be mainly caused by the altered sugars and proteins. Cellulose is one of the major structural polysaccharides in sesame. Cellulase–papain treatment may increase the accessibility of carbohydrate- and protein-derived precursors by hydrolyzing structural polysaccharides and proteins. The resulting low-molecular-weight compounds, including reducing sugars, peptides, and amino acids, may subsequently participate in Maillard-related reactions and contribute to the formation of heterocyclic volatile compounds during roasting (Ma et al., 2022; Yao et al., 2021).

As illustrated in Fig. 1B, PCA analysis was employed to elucidate the underlying correlations between the identified volatile profiles and the samples. It revealed that the first two principal components explained more than 75.2% of the total variance (49.6% for PC1 and 25.6% for PC2) in the volatile compounds. The samples treated with different enzymes and roasting temperatures cluster into three groups. The first group is in the second quadrant and comprises the NE120, CP120, HP120, PP120, and NE150 samples. The second group is in the first quadrant and comprises the CP150, HP150, PP150, and NE180 samples. The ketones, alcohols, aldehydes, and alkanes are located in this region, indicating strong correlation. CP180, HP180, and PP180 samples are in the fourth quadrant, showing strong correlation with heterocyclic compounds, such as 2,6-dimethylpyrazine, 2,5-dimethylpyrazine, and 3-phenyl-furan. This indicated that enzymatic treatment produced the same results (i.e., the same volatile compounds) with lower roasting temperatures compared to non-enzymatic treatments with higher roasting temperatures.

### 3.4 Color of sesame paste

**Fig. 2:**
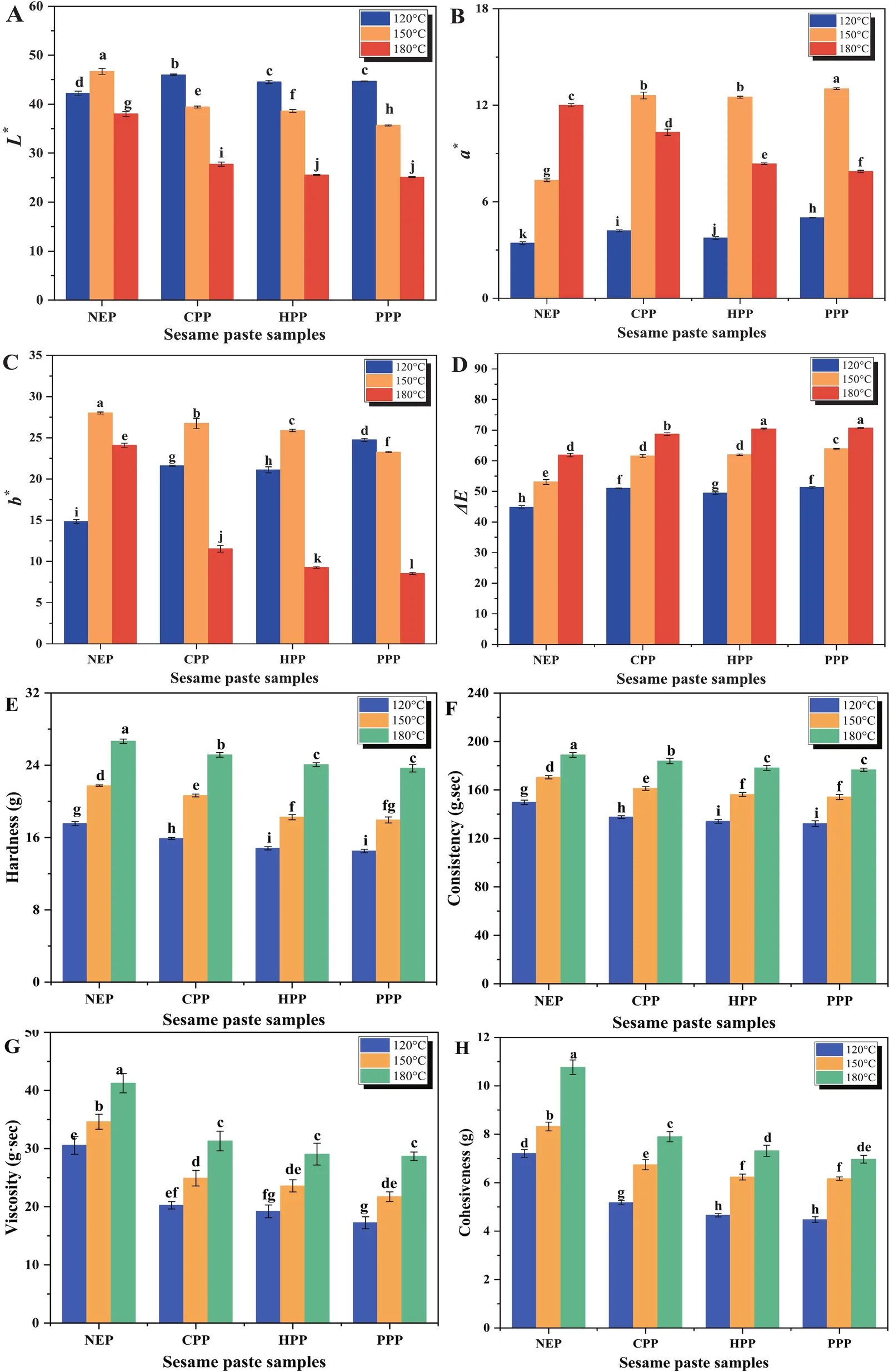
Color analysis and textural properties of twelve sesame paste samples. A, B, C, D, E, F, G, and H represent the *L*^*⁎*^ values, *a*^*⁎*^ values, *b*^*⁎*^ values, *ΔE* values, hardness, consistency, viscosity, and cohesiveness respectively. a–l indicates significant (*P* < 0.05) differences among the different treatments.

As shown in Fig. 2A, the non-enzyme-treated samples had higher *L*^*⁎*^ values than the enzyme-treated samples, and the values of *L*^*⁎*^ decreased significantly with increasing roasting temperature (*P* < 0.05), indicating that roasting and enzyme treatment could decrease the *L*^*⁎*^ value of sesame paste. This reduction in *L*^*⁎*^ value could be ascribed to the generation of brown Maillard reaction products (Mohamed Ahmed et al., 2021). As illustrated in Fig. 2B, the *a*^⁎^ values of the NEP samples were lower than those of the enzyme-treated samples at 120 °C and 150 °C. Furthermore, the *a*^⁎^ values of the enzyme-treated samples initially increased (peaking at 13.0) and subsequently decreased with increasing temperature. The *b*^*⁎*^ values also displayed an initial increase followed by a decrease with increasing temperature (Fig. 2C). Both phenomena could be attributed to the fact that the Maillard reaction proceeded only to an intermediate stage under the non-enzyme treatment and low roasting temperatures (Guo et al., 2023). The *ΔE* values (total color difference) are shown in Fig. 2D. At the same roasting temperature, CPP180, HPP180, and PPP180 exhibited higher *ΔE* values than NEP180, indicating greater overall color differences relative to the NEP group. Enzymatic hydrolysis of sesame meal has been reported to increase the availability of reducing sugars and amino-containing compounds, which may subsequently participate in Maillard reactions and produced darker-colored products with higher *ΔE* values (Ma et al., 2022). Thus, the greater color changes in the enzyme-treated samples may be associated with increased accessibility of carbohydrate- and protein-derived Maillard reaction precursors.

### 3.5 Texture profile analysis of sesame paste

Texture, an important parameter of sesame paste, may be affected by the Maillard reaction. Thus, the texture properties of all samples were assessed, and the results are presented in Fig. 2E-H. We can see that NEP samples have higher values than CPP, HPP, and PPP at the same roasting temperature. We presume this is because enzymes cause protein and structural polysaccharide degradation, disrupting the cell wall structure, which in turn affects texture (Chen et al., 2018). The texture value of the CPP sample was higher than HPP and PPP samples. Because cellulose is the main polysaccharide in sesame kernel, cellulase treatment increase the chance of the thermal reaction of sesame polysaccharides and proteins during roasting (Wen et al., 2022). In addition, the hardness, consistency, viscosity, and cohesiveness of enzymatically and non-enzymatically treated samples progressively increased with increasing roasting temperature. The reason is that high roasting temperature altered the three-dimensional structure of proteins, exposing the functional groups, and promoting the Maillard reaction (Sun et al., 2010).

### 3.6 Particle size analysis of sesame paste

**Fig. 3:**
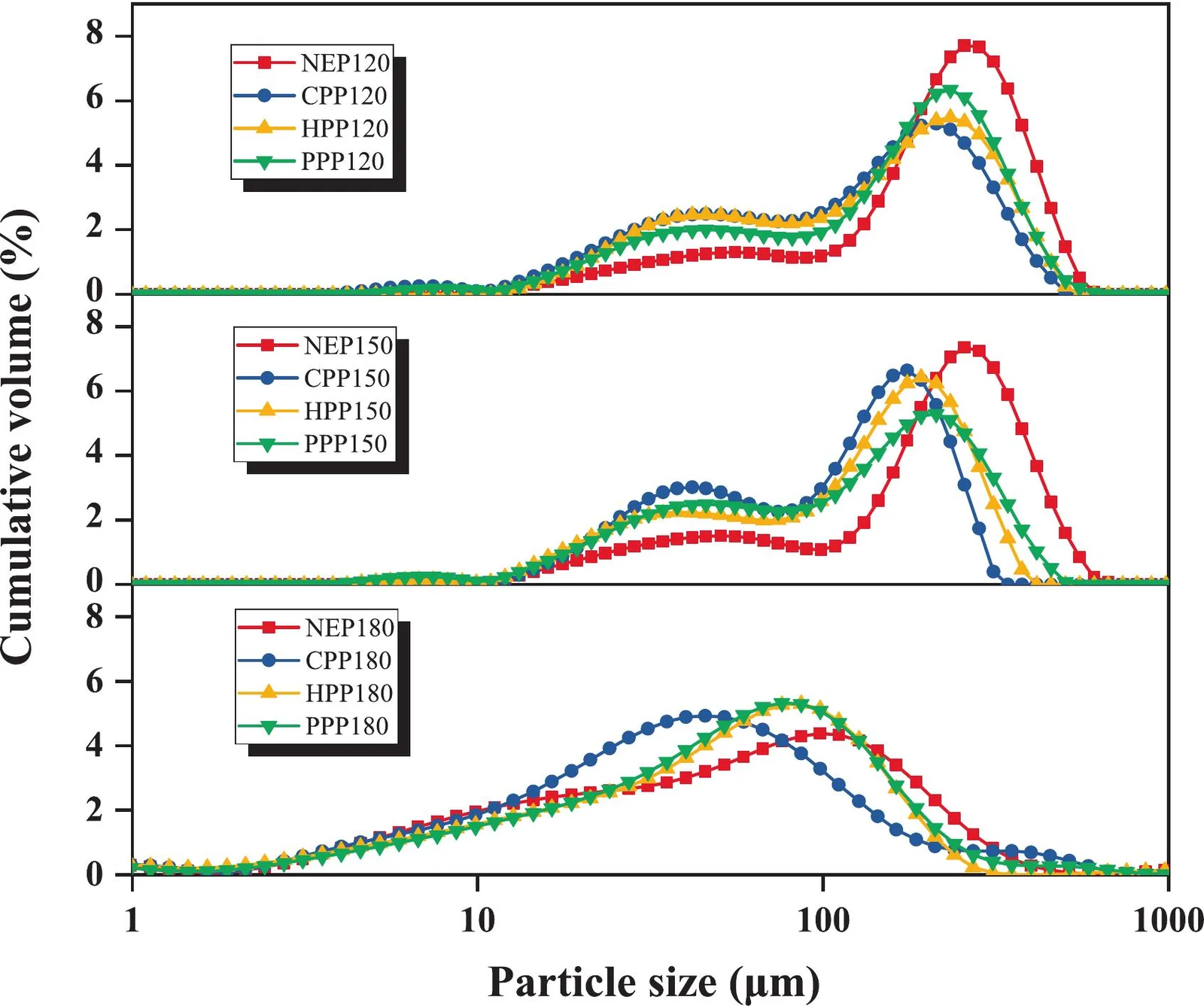
Particle size distribution of the twelve sesame paste samples. CPP, HPP, and PPP represent the paste made from sesame meal treated with cellulase-papain, hemicellulase-papain, and pectinase-papain complexes, respectively. NEP represents the paste made from sesame meal without enzyme treatment. The numbers 120, 150, and 180 indicate roasting temperatures of 120, 150, and 180 °C, respectively.

The particle size distribution of the twelve paste samples is presented in Fig. 3. Consistent with the findings of Norazatul Hanim et al. (2016), all samples displayed multimodal distributions. Under the same enzymatic treatment, as the roasting temperature increased, the volume curve of the sample shifted to the left, reflecting a diminished proportion of large particles. At the same temperature, the volume fraction curve for the CPP was on the far left and the volume fraction curve for the NEP was on the far right, indicating that enzyme treatment reduces particle size. As illustrated in Table S3, the values of D10, D50, and D90 decreased from 16.8, 144.5, and 300.7 μm for NEP120 to 7.2, 40.7, and 147.4 μm for CPP180, respectively. These results reflect the fact that enzymes decompose polysaccharides and proteins into small molecules. High roasting temperature broke intramolecular bonds and promoted the degradation of protein and polysaccharide structures, ultimately reducing particle size (He et al., 2021). Fidaleo et al. (2017) reported that sesame pastes with small particles had longer shelf life and better mouth-feel compared to those with large particles.

### 3.7 Stability analysis of sesame paste

Sesame paste is thermodynamically unstable because it is a multiphase dispersion system. The droplets and particles constantly move and collide with each other because of the effects of gravity and Brownian motion, leading to the separation of oil and solids. The oil separation rate serves as a critical indicator for assessing the colloidal stability of sesame paste.

**Fig. 4:**
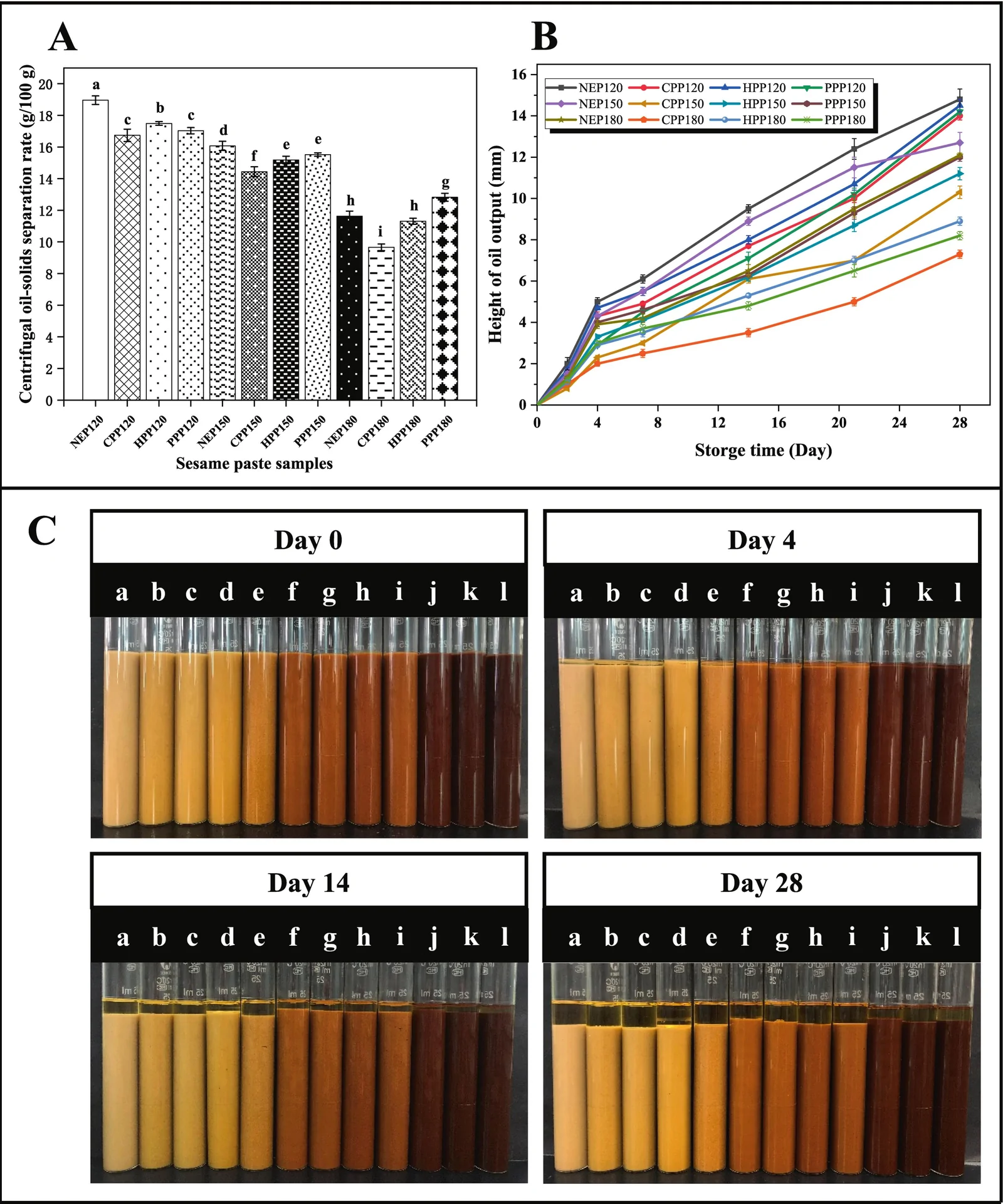
Stability of twelve sesame pastes. A represents centrifugal oil separation rate; B represents height of oil separation during storage; C represents storage change of paste. a, e, and i represent the pastes made from sesame meal without enzyme treatment roasted at 120 °C, 150 °C, and 180 °C, respectively. b, f, and j represent the pastes made from sesame meal treated with cellulase-papain complex, then roasted at 120 °C, 150 °C, and 180 °C, respectively. c, g, and k represent the pastes made from sesame meal treated with hemicellulase-papain complex then roasted at 120 °C, 150 °C, and 180 °C, respectively. d, h, and l represent pastes made from sesame meal treated with pectinase-papain complex, then roasted at 120 °C, 150 °C, and 180 °C, respectively. a–i in figure A indicates significant (*P* < 0.05) differences among the different treatments.

As can be seen in Fig. 4A, the centrifugal oil separation rate of sesame paste reduced with the increase of roasting temperature, and the sesame paste samples treated with enzymes were more stable than non-enzyme-treated samples. The CPP180 sample displayed the lowest centrifugal oil separation rate (9.66%) among all samples, indicating that the sample treated with cellulase and protein enzymes and roasted at 180 °C was the most stable.

The oil separation of all sesame paste samples during storage is shown in Fig. 4B and C. It was clear that the longer the paste sample was stored, the more oil separated out; this was especially true for samples without enzyme treatment and roasted at 120 °C.

These results were similar to the results of centrifugal oil separation rates. Several factors can explain these. Çiftçi et al. (2008) reported that decreasing particle size improved the stability of sesame paste, and suggested that small particle size improved interaction of the oil and solid phases and dispersion of the solid phase in the oil phase. Enzymatic treatments (especially cellulase-papain complex) and roasting (especially at high temperatures) can significantly reduce particle size, leading to less oil separation. This phenomenon aligns well with the result of our particle size study, in which CPP180 exhibited the smallest size. Saatchi et al. (2022) showed that enzymatic treatments and high roasting temperature promote the Maillard reaction, form polysaccharide-protein conjugates, and allow effective adsorption of particles onto the interface quickly, which reduces the oil separation capacity.

### 3.8 Rheological measurements of sesame paste

#### 3.8.1 Static rheological properties

**Fig. 5:**
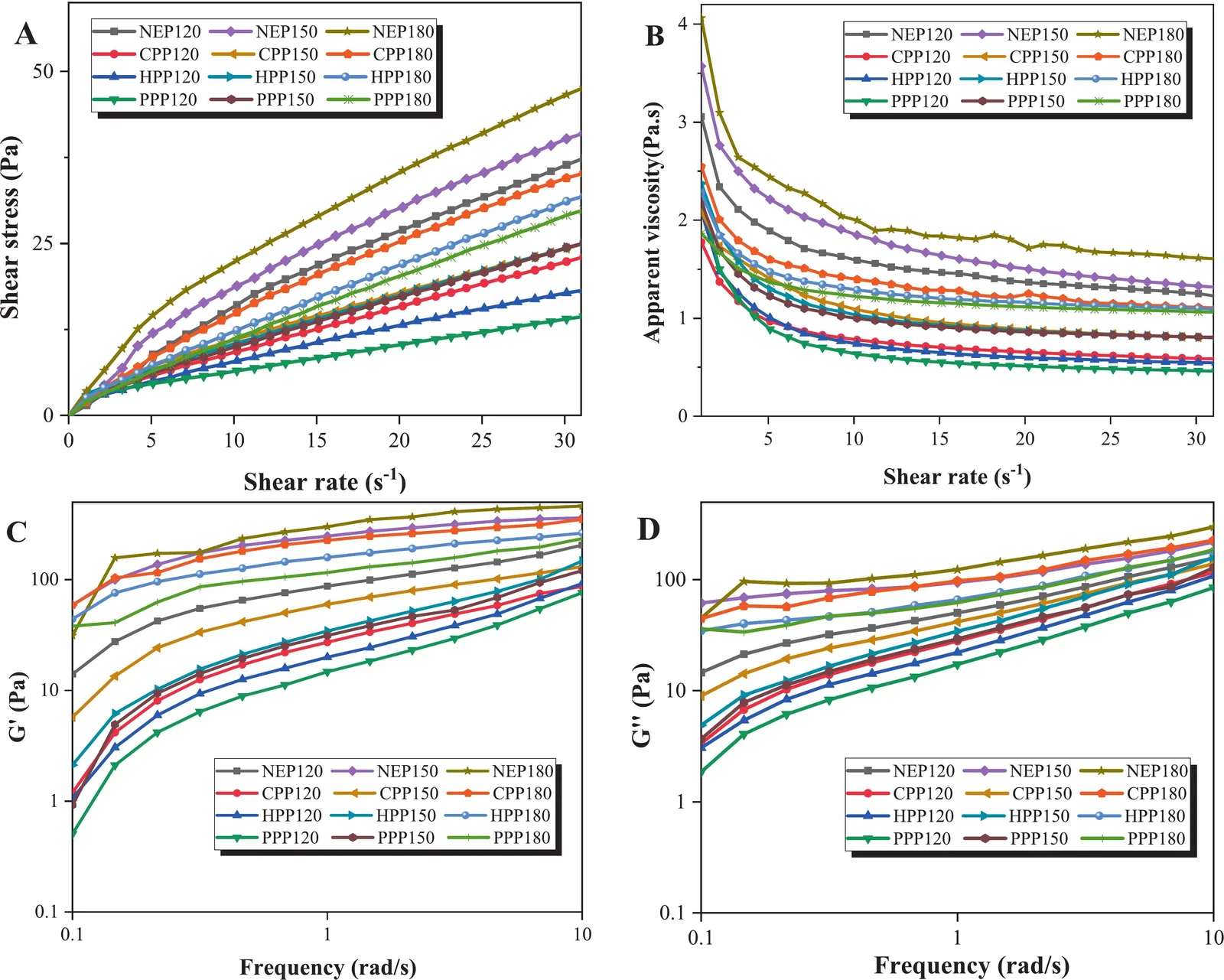
Rheological properties of the twelve sesame paste samples. G' indicates storage moduli (Pa); G" indicates loss moduli (Pa). CPP, HPP, and PPP represent the paste made from sesame meal treated with cellulase-papain, hemicellulase-papain, and pectinase-papain complexes, respectively. NEP represents the paste made from sesame meal without enzyme treatment. The numbers 120, 150, and 180 indicate roasting temperatures of 120, 150, and 180 °C, respectively.

As seen in Fig. 5A, under the same roasting condition, the shear stress of NEP was higher than CPP, HPP, and PPP. Given the same enzyme treatment, shear stress increased with rising temperature. Consistent with our observations, previous studies have demonstrated that the shear stress of flaxseed mucilage decreases with enzyme treatment (Wu et al., 2010). And Jin, Guo, et al. (2022) found that the shear stress of sesame paste increased with rising temperature. We speculate that enzymatic treatment disrupts the internal structure of the sesame meal molecules, resulting in decreased shear stress. The shear stress of CPP samples was higher than HPP and PPP samples. This may be because cellulose is the main polysaccharide in sesame meal, and the treatment with cellulase and papain enhances the interaction of small molecule proteins and polysaccharides (Liu et al., 2023; Noh et al., 2025). Another explanation is that the degree of the thermal reaction was promoted by high temperature, strengthening intermolecular interactions, forming tighter and cross-linking *net*-works between polysaccharides and proteins, leading to an increase in shear stress (Jin, Zhao, et al., 2022).

In Fig. 5B, at an identical shear rate, the PPP120 and NEP180 samples had the lowest and highest apparent viscosity among all samples, respectively. Overall, the variation of apparent viscosity of all samples displayed the same trend as shear stress. This phenomenon of decreasing apparent viscosity can be attributed to breaking of molecular entanglements, effectively reducing molecular volume. At the same time, the degree of molecular entanglement was stronger in high temperature and non-enzyme-treated samples, which may due to intermolecular interactions.

To elucidate the rheological characteristics of the paste samples, the flow data were fitted to the power-law model. As shown in supplementary materials Table S4, all the *R*^*2*^ of paste samples were over 0.98, which indicated a good fit. As a crucial indicator of flow behavior, the consistency index (K) reached its maximum and minimum values in the NEP180 and HPP120 samples, respectively. These results mean that the HPP120 and NEP180 samples were the thinnest and thickest, respectively, among all samples. Our results may be because the polysaccharides and proteins were degraded by high roasting temperature and enzyme treatment, producing small molecule peptides and sugars, improving the rate of thermal reaction, and increasing the viscosity of the sesame paste.

#### 3.8.2 Dynamic oscillatory testing

As shown in Fig. 5C and D, both the storage (*G'*) and loss (*G"*) increased with frequency. For all paste samples, *G'* values were consistently below *G"* at identical frequencies, meaning that they exhibited liquid-like behavior. The NEP180 and PPP120 samples had the highest and lowest *G'* values, respectively, among all samples, and the trend of the *G"* values was similar to *G'* values. Firstly, cellulase and papain can degrade more small molecules sugars and proteins, which facilitated the Maillard reaction and produced stable network-type structures and strong particle-particle interactions (Vandecan & Daems, 2011). Secondly, high temperature promoted the reaction between the hydroxyl groups of small molecule sugars and the amino groups of proteins, forming protein-polysaccharide conjugates, enhancing the intermolecular interaction force, leading to an increase in *G'* and *G"* values. Therefore, enzyme treatment could increase the thermal reaction between proteins and polysaccharides, facilitating the formation of tighter cross-linking networks, which play an important role in maintaining the semisolid strength of sesame paste (Miao et al., 2022).

### 3.9 Microstructure of sesame paste

The proteins and lipids of sesame paste samples were stained with FITC and Nile red to observe the microstructure of the twelve pastes using CLSM. As seen in supplementary materials Fig. S2, the proteins of NEP samples were in a dispersed state compared with CPP, HPP, and PPP samples, which indicated that enzyme treatment caused the internal structure of the protein to unfold and enabled increased protein crosslinking during roasting (Achouri & Boye, 2013). The dispersed state of proteins increased as roasting temperature decreased. This can be explained that the thermal reaction between proteins and polysaccharides produced polysaccharide-protein conjugates, enhancing oil adsorption capacity and cohesiveness of the protein network. The green area of CPP180 was more extensive and showed more protein-polysaccharide cross-linking compared to the other samples. This may be because cellulose is the major polysaccharide, and the high temperature and enzymatic treatment promoted polysaccharide-protein reactions (Liu et al., 2023). These results are consistent with stability analysis of sesame paste and previous reports (Hatamian et al., 2020).

## 4 Conclusion

This study aimed to develop a novel strategy for producing high-quality and low-hazard sesame paste by utilizing enzymatically hydrolyzed sesame meal as the raw material. The results showed that the cellulase–papain treatment reduced the total amino acid and crude polysaccharide contents of the roasted sesame meal and increased its total volatile compound content. The corresponding sesame paste exhibited a greater total color difference, a smaller apparent particle size, and improved colloidal stability, as evidenced by a lower centrifugal oil separation rate. Notably, roasting at 180 °C improved the texture and stability of sesame paste, but it concurrently elevated the concentrations of norharman and harman. In comparison, the enzymatically treated sample roasted at 150 °C (CPP150) exhibited texture, stability, and volatile compound profiles comparable to those of the non-enzymatic sample roasted at 180 °C (NEP180), while containing significantly lower concentrations of the two quantified β-carboline heterocyclic amines. These findings indicate that enzymatic pretreatment can achieve desirable color, flavor and texture properties in sesame paste at a lower roasting temperature and simultaneously limit the formation of norharman and harman. This processing strategy provides a reference for optimizing the quality and processing conditions of sesame paste products.

## CRediT authorship contribution statement

**Lei Jin:** Writing – original draft, Software, Methodology, Investigation, Formal analysis, Data curation, Conceptualization. **Yu-Xiang Ma:** Methodology. **Li-Xia Hou:** Software. **Xue-De Wang:** Investigation. **Jin-chu Yang:** Supervision, Resources. **Yong-ming Xu:** Supervision, Resources. **Hua-Min Liu:** Writing – review & editing, Supervision, Resources.

## Declaration of competing interest

The authors declare that they have no known competing financial interests or personal relationships that could have appeared to influence the work reported in this paper.

## Data Availability

Data will be made available on request.

## Appendix A Supplementary data

Supplementary material: Low-temperature preparation of enzyme-treated sesame paste: Quality enhancement and hazard mitigation.
