# Supplementary material for: Low-temperature preparation of enzyme-treated sesame paste: Quality enhancement and hazard mitigation
Source: Food Chem X. 2026 Aug 29;39:104341. doi: 10.1016/j.fochx.2026.104341 (PMC13562424; doi:10.1016/j.fochx.2026.104341)
Supplement: Supplementary file 1 [file mmc1.docx]

**Supplementary data**

**Low-temperature preparation of enzyme-treated sesame paste: Quality enhancement and hazard mitigation**

Lei Jin^a^, Yu-Xiang Ma^a^, Li-Xia Hou^a^, Xue-De Wang^a^, Jin-chu Yang^b^, Yong-ming Xu^b^, Hua-Min Liu^a^*^*^*

^a^ *College of Food Science and Technology & Institute of Special Oilseed Processing and Technology, Henan University of Technology, Zhengzhou 450001, China*

^b^ *Technology Center, China Tobacco Henan Industrial Co., Ltd., Zhengzhou 450000, China*

*^*^***Corresponding authors:** College of Food Science and Technology, No.100 Lotus Street, High-tech Zone, Zhengzhou 450001, China.

**E-mail address:** liuhuamin5108@163.com (Hua-Min Liu).

**Table S1** Chemical components and HAs concentration of the all sesame meal.

| Sample* | Protein  (g/100 g) | Moisture  (g/100 g) | Fat  (g/100 g) | Crude polysaccharide  (g/100 g) | Norharman (ng/mL) | Harman (ng/mL) |
| --- | --- | --- | --- | --- | --- | --- |
| NE120 | 47.04±0.35^a^ | 1.75±0.09^a^ | 1.29±0.12^d^ | 16.62±0.25^a^ | 1.32±0.09^d^ | < LOD |
| CP120 | 45.34±0.22^cd^ | 1.72±0.07^a^ | 1.69±0.06^c^ | 15.37±0.19^c^ | 0.45±0.04^d^ | < LOQ |
| HP120 | 45.71±0.54^bc^ | 1.73±0.04^a^ | 1.70±0.11^c^ | 15.97±0.09^b^ | 0.73±0.08^d^ | < LOD |
| PP120 | 46.13±0.49^b^ | 1.80±0.11^a^ | 1.62±0.10^c^ | 15.82±0.21^b^ | 1.22±0.05^d^ | < LOD |
| NE150 | 45.24±0.67^cd^ | 1.32±0.12^b^ | 1.58±0.06^c^ | 15.34±0.27^c^ | 2.69±0.14^d^ | 0.49±0.03^e^ |
| CP150 | 43.05±0.19^e^ | 1.28±0.08^b^ | 1.94±0.14^b^ | 14.13±0.36^d^ | 4.44±0.21^d^ | 0.95±0.04^e^ |
| HP150 | 43.35±0.15^e^ | 1.31±0.05^b^ | 2.05±0.05^b^ | 14.28±0.34^d^ | 4.06±0.11^d^ | 0.73±0.04^e^ |
| PP150 | 44.86±0.27^d^ | 1.39±0.03^b^ | 1.90±0.09^b^ | 14.53±0.19^d^ | 4.44±0.18^d^ | 0.82±0.03^e^ |
| NE180 | 43.63±0.32^e^ | 1.12±0.10^c^ | 2.03±0.10^b^ | 13.59±0.26^e^ | 37.95±1.62^c^ | 5.86±0.18^d^ |
| CP180 | 40.14±0.11^g^ | 0.95±0.02^c^ | 2.50±0.13^a^ | 11.81±0.06^g^ | 62.74±2.33^b^ | 22.93±0.92^b^ |
| HP180 | 40.72±0.24^g^ | 1.02±0.06^c^ | 2.44±0.08^a^ | 12.38±0.22^f^ | 78.89±3.71^a^ | 27.11±0.84^a^ |
| PP180 | 42.23±0.18^f^ | 0.99±0.07^c^ | 2.31±0.07^a^ | 12.21±0.13^f^ | 66.07±2.07^b^ | 15.34±0.67^c^ |

*^*^*NE represents sesame meal without enzyme treatment; CP, HP, and PP represent sesame meal enzymatically treated by cellulase-papain complex, hemicellulase-papain complex, and pectinase-papain complex, respectively. Numbers 120, 150, 180 correspond to the sesame meal roasted at 120°C, 150°C, and 180°C, respectively. LOD indicates that the analyte concentration was below the limit of detection; LOQ indicates that the analyte concentration was below the limit of quantification.

**Table S2** Volatile compounds in twelve sesame meal samples (ND, not detected).

| NO | Volatile compounds | RI | IDEN | Content (ug/kg) | | | | | | | | | | | |
| --- | --- | --- | --- | --- | --- | --- | --- | --- | --- | --- | --- | --- | --- | --- | --- |
|  |  |  |  | NE120*^*^* | CP120 | HP120 | PP120 | NE150 | CP150 | HP150 | PP150 | NE180 | CPP180 | HP180 | PP180 |
|  | **Aldehydes** |  |  |  |  |  |  |  |  |  |  |  |  |  |  |
| 1 | 3-methyl-Butanal | 703 | MS | ND | 1.51 | ND | 5.27 | 4.81 | 68.65 | 33.44 | 21.83 | ND | 24.11 | 11.16 | ND |
| 2 | Pentanal | 706 | MS,RI | ND | 5.26 | ND | ND | 1.47 | ND | ND | ND | ND | ND | ND | ND |
| 3 | Benzaldehyde | 954 | MS,RI | 0.87 | 17.68 | 12.71 | 8.24 | 16.58 | 50.46 | 57.40 | 52.19 | 43.41 | 38.03 | 37.94 | 44.93 |
| 4 | Benzeneacetaldehyde | 1040 | MS,RI | ND | 3.39 | ND | 0.67 | ND | 3.61 | ND | 5.04 | ND | ND | ND | ND |
| 5 | Nonanal | 1100 | MS,RI | 0.44 | 0.55 | ND | 0.20 | 0.80 | ND | ND | ND | 5.38 | ND | ND | ND |
| 6 | 5-Methyl-2-phenyl-2-hexenal | 1505 | MS | ND | 1.14 | ND | 0.55 | ND | 1.63 | 1.60 | 2.11 | ND | ND | ND | ND |
| 7 | Hexanal | 779 | MS,RI | 0.08 | ND | ND | ND | 2.75 | ND | 0.18 | ND | 12.99 | 0.87 | 1.03 | 0.96 |
| 8 | Phenylglyoxal | 954 | MS | 6.78 | ND | ND | ND | ND | ND | ND | ND | ND | ND | ND | ND |
| 9 | 2-butyl-2-Octenal | 1379 | MS | 0.14 | ND | ND | ND | ND | ND | ND | ND | ND | ND | ND | ND |
| 10 | Furfural | 832 | MS,RI | ND | ND | ND | ND | 11.17 | 40.17 | 40.91 | 30.17 | ND | 21.17 | 25.91 | 17.17 |
| 11 | 2-Isopropyl-5-methylhex-2-enal | 1107 | MS | ND | ND | ND | ND | ND | 1.33 | 0.94 | 1.84 | ND | ND | 0.44 | 0.41 |
| 12 | 4,4-Dimethylpent-2-enal | 862 | MS | ND | ND | ND | ND | ND | ND | 0.29 | 0.59 | ND | ND | ND | ND |
| 13 | Propanal | 987 | MS,RI | ND | ND | ND | ND | ND | ND | 0.17 | ND | ND | ND | ND | ND |
| 14 | 2-methyl-Butanal | 655 | MS | ND | ND | ND | ND | 1.76 | ND | ND | ND | ND | ND | ND | ND |
| 15 | Heptanal | 864 | MS,RI | ND | ND | ND | ND | 0.26 | ND | ND | ND | 1.77 | ND | ND | ND |
| 16 | Octanal | 985 | MS,RI | ND | ND | ND | ND | 0.24 | ND | ND | ND | 2.27 | ND | ND | ND |
| 17 | 5-Ethylcyclopent-1-enecarboxaldehyde | 1015 | MS | ND | ND | ND | ND | 0.31 | ND | ND | ND | ND | ND | ND | ND |
| 18 | 3,3-Diethoxy-1-propyne | 826 | MS | ND | ND | ND | ND | ND | ND | ND | ND | 0.11 | ND | ND | ND |
| 19 | (E)-2-Heptenal | 916 | MS,RI | ND | ND | ND | ND | ND | ND | ND | ND | 3.93 | ND | ND | ND |
| 20 | (E)-2-Octenal | 1035 | MS,RI | ND | ND | ND | ND | ND | ND | ND | ND | 0.76 | ND | ND | ND |
| 21 | Decanal | 1193 | MS,RI | ND | ND | ND | ND | ND | ND | ND | ND | 0.35 | ND | ND | ND |
| 22 | (Z)-2-Decenal | 1252 | MS,RI | ND | ND | ND | ND | ND | ND | ND | ND | 2.40 | ND | ND | ND |
| 23 | 2,4-Decadienal | 1287 | MS,RI | ND | ND | ND | ND | ND | ND | ND | ND | 6.55 | ND | ND | ND |
| 24 | (E,E)-2,4-Decadienal | 1310 | MS,RI | ND | ND | ND | ND | ND | ND | ND | ND | 29.91 | ND | ND | ND |
| 25 | 2-Undecenal | 1356 | MS,RI | ND | ND | ND | ND | ND | ND | ND | ND | 2.52 | ND | ND | ND |
|  | Total |  |  | 8.32 | 29.54 | 12.71 | 14.93 | 40.13 | 165.85 | 134.94 | 113.77 | 112.36 | 84.18 | 76.48 | 63.48 |
|  | **Ketones** |  |  |  |  |  |  |  |  |  |  |  |  |  |  |
| 26 | Acetophenone | 1065 | MS,RI | ND | 0.72 | ND | ND | 2.65 | 13.86 | 4.95 | 8.30 | ND | 3.29 | ND | 3.14 |
| 27 | 4-Nonanone | 1068 | MS | 0.96 | 0.59 | 0.36 | 0.34 | ND | ND | ND | ND | ND | ND | ND | ND |
| 28 | Acetone | 682 | MS,RI | ND | ND | ND | ND | ND | 1.99 | 2.37 | 2.99 | 1.91 | ND | 0.40 | 0.90 |
| 29 | 2-Butanone | 687 | MS,RI | ND | ND | ND | ND | 0.24 | 13.51 | 19.34 | 4.17 | 12.42 | ND | ND | ND |
| 30 | Acetoin | 698 | MS,RI | ND | ND | ND | ND | ND | ND | 4.11 | ND | ND | ND | ND | ND |
| 31 | 4,6-dimethyl-2,7-Nonadien-5-one | 862 | MS | ND | ND | ND | ND | ND | ND | ND | 0.24 | ND | ND | ND | ND |
| 32 | 2-Decanone | 1194 | MS,RI | ND | ND | ND | ND | ND | ND | ND | 0.53 | ND | ND | ND | ND |
| 33 | 2,2,3-trimethyl-Cyclobutanone | 688 | MS | ND | ND | ND | ND | ND | ND | ND | ND | ND | ND | ND | 0.46 |
| 34 | 5-methyl-1-phenyl-1-Hexanone | 1415 | MS | ND | ND | ND | ND | ND | ND | ND | ND | ND | ND | 0.52 | 0.56 |
| 35 | 2,3-Hexanedione | 707 | MS | ND | ND | ND | ND | ND | ND | ND | ND | ND | ND | 0.09 | ND |
| 36 | Propyl pyruvate | 845 | MS | ND | ND | ND | ND | ND | ND | ND | ND | ND | ND | 0.13 | ND |
| 37 | 1-phenyl-1-Hexanone | 1415 | MS | ND | ND | ND | ND | ND | ND | ND | ND | ND | ND | 0.61 | ND |
| 38 | 3-Octen-2-one | 1015 | MS | ND | ND | ND | ND | ND | ND | ND | ND | 1.46 | ND | ND | ND |
| 39 | 3-Nonen-2-one | 1124 | MS | ND | ND | ND | ND | ND | ND | ND | ND | 2.37 | ND | ND | ND |
|  | Total |  |  | 0.96 | 1.31 | 0.36 | 0.34 | 2.89 | 29.36 | 30.76 | 16.24 | 18.16 | 3.29 | 1.76 | 5.06 |
|  | **Pyrazines** |  |  |  |  |  |  |  |  |  |  |  |  |  |  |
| 40 | 2,5-dimethyl-Pyrazine | 906 | MS,RI | ND | 7.46 | 4.39 | 3.15 | ND | 2.81 | 2.06 | 1.41 | 3.12 | 51.39 | 31.92 | 24.01 |
| 41 | 2-ethyl-5-methyl-Pyrazine | 998 | MS | ND | 1.62 | 1.07 | 0.76 | ND | ND | ND | ND | 2.79 | 5.79 | 10.05 | ND |
| 42 | 2-Isoamyl-6-methylpyrazine | 1250 | MS | ND | 0.34 | ND | ND | ND | 3.40 | 4.52 | 2.48 | 1.84 | 7.59 | 4.51 | 4.99 |
| 43 | 2-(3-Methylbutyl)-3,5-dimethylpyrazine | 1317 | MS | ND | 0.90 | 0.30 | ND | ND | ND | ND | 4.30 | ND | ND | ND | ND |
| 44 | 2,3-Dimethyl-5-isopentylpyrazine | 1317 | MS | ND | 0.90 | ND | ND | ND | ND | ND | ND | ND | ND | ND | ND |
| 45 | 3-ethyl-2,5-dimethyl-Pyrazine | 1076 | MS,RI | ND | ND | 0.35 | 0.24 | 0.28 | 3.16 | 3.85 | 2.35 | 0.39 | 6.39 | 3.55 | 2.91 |
| 46 | 2,6-diethyl-Pyrazine | 1076 | MS | ND | ND | 0.35 | ND | ND | 0.75 | ND | ND | ND | ND | ND | ND |
| 47 | methyl-Pyrazine | 819 | MS | ND | ND | ND | ND | ND | ND | 1.53 | 2.07 | 5.28 | 24.38 | 17.65 | 13.25 |
| 48 | 2-ethyl-6-methyl-Pyrazine | 1001 | MS | ND | ND | ND | ND | ND | ND | 0.73 | ND | ND | 16.82 | ND | 8.59 |
| 49 | trimethyl-Pyrazine | 1003 | MS,RI | ND | ND | ND | ND | ND | ND | ND | 1.40 | 1.16 | 11.57 | 7.70 | 3.71 |
| 50 | 3,5-Dimethyl-2-isobutylpyrazine | 1207 | MS | ND | ND | ND | ND | ND | ND | ND | ND | ND | 0.68 | ND | 0.35 |
| 51 | 2,5-dimethyl-3-(3-methylbutyl)-Pyrazine | 1322 | MS | ND | ND | ND | ND | ND | 7.51 | 9.00 | ND | 3.48 | 13.33 | 6.69 | 7.43 |
| 52 | 2,3,5-Trimethyl-6-isopentylpyrazine | 1394 | MS | ND | ND | ND | ND | ND | 0.74 | ND | ND | ND | 1.76 | 0.74 | 0.94 |
| 53 | 5H-5-Methyl-6,7-dihydrocyclopentapyrazine | 1123 | MS | ND | ND | ND | ND | ND | ND | ND | 0.24 | ND | ND | ND | ND |
| 54 | 2,3-dimethyl-Pyrazine | 869 | MS | ND | ND | ND | ND | ND | ND | 0.44 | ND | 2.06 | ND | ND | ND |
| 55 | 2,6-dimethyl-Pyrazine | 860 | MS,RI | ND | ND | ND | ND | ND | 4.00 | ND | ND | ND | ND | ND | ND |
| 56 | 6,7-dihydro-2,5-dimethyl-5H-Cyclopentapyrazine | 1225 | MS | ND | ND | ND | ND | ND | ND | ND | ND | ND | 1.27 | ND | ND |
|  | Total |  |  | ND | 11.22 | 6.47 | 4.15 | 0.28 | 22.36 | 22.14 | 14.26 | 20.12 | 140.97 | 82.81 | 66.18 |
|  | **Furans** |  |  |  |  |  |  |  |  |  |  |  |  |  |  |
| 57 | 2-pentyl-Furan | 984 | MS | 0.87 | 1.44 | 0.89 | 1.00 | 2.71 | 5.61 | 3.20 | 3.13 | 6.29 | 23.69 | 4.54 | 4.47 |
| 58 | Furfurylmethylamphetamine | 983 | MS | ND | ND | ND | 1.00 | ND | ND | ND | ND | ND | 0.77 | ND | ND |
| 59 | 3-phenyl-Furan | 1229 | MS | ND | ND | ND | ND | 0.59 | 1.57 | 2.25 | 1.43 | ND | ND | 2.46 | 1.78 |
| 60 | N-(2-furanylmethylene)-3-methyl-1-Butanamine | 1295 | MS | ND | ND | ND | ND | ND | 0.61 | 0.59 | ND | ND | ND | ND | 1.63 |
| 61 | 2(3H)-Furanone, 5-acetyldihydro-2(3H)-Furanone | 780 | MS | ND | ND | ND | ND | ND | ND | ND | ND | ND | ND | 0.11 | ND |
| 62 | 1-(2-furanyl)-Ethanone | 906 | MS | ND | ND | ND | ND | ND | 4.87 | ND | ND | ND | ND | 9.64 | 2.17 |
| 63 | 2-n-Butyl furan | 828 | MS,RI | ND | ND | ND | ND | ND | ND | ND | ND | ND | 0.30 | ND | ND |
|  | Total |  |  | 0.87 | 1.44 | 0.89 | 2.00 | 3.29 | 12.66 | 6.04 | 4.56 | 6.29 | 24.76 | 16.75 | 10.05 |
|  | **Pyridines** |  |  |  |  |  |  |  |  |  |  |  |  |  |  |
| 64 | 2-(2-Methylpropyl)-3,5-di(1-methylethyl) pyridine | 1493 | MS | ND | ND | ND | ND | 0.27 | 4.06 | 0.30 | ND | ND | 0.49 | 1.22 | ND |
| 65 | 4-Isopropylpyridine | 1020 | MS | ND | ND | ND | ND | ND | ND | 2.32 | ND | ND | ND | ND | ND |
| 66 | 2-hexyl-Pyridine | 1199 | MS | ND | ND | ND | ND | ND | ND | 1.12 | ND | ND | ND | ND | ND |
| 67 | 5-methyl-2-Pyridinamine | 885 | MS | ND | ND | ND | ND | ND | ND | ND | 2.17 | ND | 0.70 | ND | ND |
| 68 | 2,4(1H,3H)-dione, 5-amino-6-nitroso-Pyrimidine | 678 | MS | ND | ND | ND | ND | ND | 0.31 | ND | ND | ND | ND | ND | ND |
| 69 | 2-(2-methylpropyl)-Pyridine | 1186 | MS | ND | ND | ND | ND | ND | ND | ND | ND | 3.95 | ND | ND | ND |
| 70 | 2-propyl-Pyridine | 1185 | MS | ND | ND | ND | ND | ND | ND | ND | ND | ND | 11.47 | ND | ND |
| 71 | 2-pentyl-Pyridine | 1185 | MS | ND | ND | ND | ND | ND | ND | ND | ND | ND | 11.95 | ND | 8.93 |
| 72 | 2-butyl-Pyridine | 1185 | MS | ND | ND | ND | ND | ND | ND | ND | ND | ND | ND | 8.65 | ND |
|  | Total |  |  | ND | ND | ND | ND | 0.27 | 4.37 | 3.74 | 2.17 | 3.95 | 24.61 | 9.87 | 8.93 |
|  | **Alcohols** |  |  |  |  |  |  |  |  |  |  |  |  |  |  |
| 73 | Cyclopropyl methyl carbinol | 703 | MS | ND | 1.17 | ND | ND | ND | ND | ND | ND | ND | ND | ND | ND |
| 74 | (2-Methyloxan-2-yl) methanol | 1068 | MS | 0.71 | 0.25 | 0.71 | 0.47 | ND | ND | ND | ND | ND | ND | ND | ND |
| 75 | Methanethiol | 679 | MS | ND | ND | ND | ND | ND | 0.57 | 0.42 | 0.58 | ND | ND | ND | ND |
| 76 | 1-Pentanol | 709 | MS,RI | ND | ND | ND | ND | 0.86 | ND | ND | ND | 4.69 | 0.13 | 0.24 | ND |
| 77 | (1R,2S) -(-)-2-Amino-1,2-diphenylethanol | 995 | MS | ND | ND | ND | ND | ND | ND | ND | ND | ND | 0.49 | ND | ND |
| 78 | O-(4-butylbenzoyl)-O'-(isobutoxycarbonyl)-1,2-Benzenediol | 1351 | MS | ND | ND | ND | ND | ND | ND | ND | ND | ND | ND | ND | 0.14 |
| 79 | 1-Octen-3-ol | 950 | MS,RI | ND | ND | ND | ND | ND | ND | ND | ND | 3.96 | ND | ND | ND |
| 80 | (S)-3-Ethyl-4-methylpentanol | 1054 | MS | ND | ND | ND | ND | ND | ND | ND | ND | 1.24 | ND | ND | ND |
|  | Total |  |  | 0.71 | 1.42 | 0.71 | 0.47 | 0.86 | 0.57 | 0.42 | 0.58 | 9.89 | 0.62 | 0.24 | 0.14 |
|  | **Esters** |  |  |  |  |  |  |  |  |  |  |  |  |  |  |
| 81 | Nonanoic acid, ethyl ester | 1296 | MS | 0.84 | 0.82 | ND | 0.45 | ND | ND | 0.79 | ND | ND | ND | ND | ND |
| 82 | Hexadecanoic acid, ethyl ester | 2027 | MS | 4.22 | 4.01 | 2.53 | 4.60 | 2.78 | 1.86 | 3.08 | ND | 0.43 | ND | 0.69 | 0.39 |
| 83 | Isophthalic acid, di(2-isopropylphenyl) ester | 1116 | MS | ND | ND | ND | 0.16 | ND | ND | ND | ND | ND | 0.11 | ND | ND |
| 84 | (E)-9-Octadecenoic acid ethyl ester | 2206 | MS | 3.99 | ND | ND | 5.80 | 2.65 | ND | 2.22 | ND | ND | ND | ND | ND |
| 85 | Oxalic acid, diallyl ester | 708 | MS | ND | ND | 6.43 | ND | ND | ND | ND | ND | 2.66 | ND | ND | 0.58 |
| 86 | Methyl nitrite | 707 | MS | ND | ND | ND | ND | ND | 0.06 | ND | ND | ND | ND | ND | ND |
| 87 | Acetic acid, 2-(methylaminoethyl) ester | 678 | MS | ND | ND | ND | ND | 0.40 | ND | 0.35 | ND | ND | ND | ND | ND |
| 88 | n-Propyl acetate | 695 | MS | ND | ND | ND | ND | 1.34 | ND | ND | ND | ND | ND | ND | ND |
| 89 | Formic acid, 2-propenyl ester | 706 | MS | ND | ND | ND | ND | 2.60 | ND | ND | ND | ND | ND | ND | ND |
| 90 | Carbonic acid, ethyl 2-propenyl ester | 711 | MS | ND | ND | ND | ND | 1.22 | ND | ND | ND | ND | ND | ND | ND |
| 91 | 4-Ethylbenzoic acid, tridec-2-ynyl ester | 1225 | MS | ND | ND | ND | ND | 0.75 | ND | ND | ND | ND | ND | ND | ND |
| 92 | Hydrogen isocyanate | 679 | MS | ND | ND | ND | ND | ND | ND | 0.32 | ND | ND | ND | ND | ND |
| 93 | Oxalic acid, allyl isobutyl ester | 708 | MS | ND | ND | ND | ND | ND | ND | ND | 1.01 | ND | ND | ND | ND |
| 94 | Formic acid, pentyl ester | 709 | MS | ND | ND | ND | ND | ND | ND | ND | 0.86 | ND | ND | ND | ND |
| 95 | Benzoyl isothiocyanate | 960 | MS | ND | ND | ND | ND | ND | ND | ND | 1.78 | ND | ND | 1.26 | ND |
| 96 | Methane, isocyanato | 1602 | MS | ND | ND | ND | ND | ND | ND | ND | ND | 0.13 | ND | ND | ND |
|  | Total |  |  | 9.04 | 4.83 | 8.96 | 11.00 | 12.74 | 1.92 | 6.75 | 3.65 | 3.23 | 0.11 | 1.95 | 0.98 |
|  | **Pyrroles** |  |  |  |  |  |  |  |  |  |  |  |  |  |  |
| 97 | 3-Formyl-4,5-dimethyl-pyrrole | 1164 | MS | ND | ND | ND | ND | ND | 0.55 | ND | ND | ND | ND | ND | ND |
| 98 | 1-methyl-2-carboxaldehyde-1H-Pyrrole | 1003 | MS | ND | ND | ND | ND | ND | ND | ND | 1.02 | ND | ND | ND | ND |
| 99 | 2-methyl-1H-Pyrrole | 1048 | MS | ND | ND | ND | ND | ND | ND | 0.59 | 0.06 | ND | 0.61 | ND | 4.01 |
| 100 | 1,5-Dimethyl-2-pyrrolecarbonitrile | 1108 | MS | ND | ND | ND | ND | ND | ND | 0.80 | ND | ND | ND | ND | ND |
| 101 | 2-methyl-3-carbonitrile-(1H) Pyrrole | 935 | MS | ND | ND | ND | ND | ND | ND | ND | ND | ND | 12.23 | ND | ND |
| 102 | 2-carboxaldehyde-1H-Pyrrole | 1011 | MS,RI | ND | ND | ND | ND | ND | ND | ND | ND | 0.98 | ND | 8.27 | ND |
|  | Total |  |  | ND | ND | ND | ND | ND | 0.55 | 1.39 | 1.08 | 0.98 | 12.84 | 8.27 | 4.01 |
|  | **Phenols** |  |  |  |  |  |  |  |  |  |  |  |  |  |  |
| 103 | Butylated Hydroxytoluene | 1533 | MS | ND | ND | ND | ND | 0.18 | ND | ND | ND | ND | ND | ND | ND |
| 104 | Maltol | 1122 | MS,RI | ND | ND | ND | ND | ND | 8.55 | 7.91 | 5.01 | ND | 3.52 | 0.80 | ND |
| 105 | monobenzoate-1,3-Benzenediol | 1306 | MS | ND | ND | ND | ND | ND | 0.49 | ND | ND | ND | ND | ND | 1.07 |
| 106 | 4'-Hydroxypalmitophenone | 1390 | MS | ND | ND | ND | ND | ND | ND | 1.80 | ND | ND | ND | ND | ND |
| 107 | Hexylresorcinol | 1412 | MS | ND | ND | ND | ND | ND | ND | ND | ND | 0.10 | ND | ND | ND |
|  | Total |  |  | ND | ND | ND | ND | 0.18 | 9.04 | 9.70 | 5.01 | 0.10 | 3.52 | 0.80 | 1.07 |
|  | **Acids** |  |  |  |  |  |  |  |  |  |  |  |  |  |  |
| 108 | Acetic acid | 698 | MS,RI | ND | 5.92 | ND | ND | ND | ND | ND | ND | ND | ND | ND | ND |
| 109 | 1-cyclopenten-3-on-1-ylester-2,4-Pentadienoic acid | 983 | MS | ND | ND | 0.89 | ND | ND | ND | ND | ND | ND | ND | ND | ND |
| 110 | 4-Benzyloxy-3-methoxy-2-nitrobenzoic acid | 752 | MS | 1.25 | ND | ND | ND | ND | ND | ND | ND | ND | ND | ND | ND |
| 111 | Ammonium acetate | 699 | MS | ND | ND | ND | 2.68 | ND | ND | ND | ND | ND | ND | ND | ND |
|  | Total |  |  | 1.25 | 5.92 | 0.89 | 2.68 | ND | ND | ND | ND | ND | ND | ND | ND |
|  | **Alkanes** |  |  | ND | ND | ND | ND | ND | ND | ND | ND | ND | ND | ND | ND |
| 112 | Neopentane | 709 | MS,RI | ND | 1.03 | 0.24 | ND | ND | 3.76 | ND | ND | ND | ND | ND | ND |
| 113 | Tetradecane | 1398 | MS,RI | 0.20 | 0.22 | ND | ND | ND | ND | ND | ND | ND | ND | ND | ND |
| 114 | Hexadecane | 1612 | MS | 0.22 | 0.20 | ND | 0.13 | ND | ND | 0.20 | ND | 0.15 | ND | ND | ND |
| 115 | 2,6-dimethyl-Heptadecane | 1612 | MS | ND | 0.20 | ND | ND | ND | ND | ND | ND | ND | ND | ND | ND |
| 116 | 4,7-dimethyl-Undecane | 1397 | MS | ND | ND | ND | 0.15 | ND | ND | ND | ND | 2.64 | 0.13 | ND | 0.58 |
| 117 | Isobutane | 702 | MS | ND | ND | 9.37 | ND | ND | ND | ND | ND | ND | ND | ND | ND |
| 118 | 4-methyl-Undecane | 1397 | MS | ND | ND | 0.11 | ND | ND | ND | ND | ND | ND | ND | ND | ND |
| 119 | Dodecane | 1201 | MS,RI | 0.11 | ND | ND | ND | ND | ND | ND | ND | ND | ND | ND | ND |
| 120 | Trimethylene oxide | 712 | MS | ND | ND | ND | ND | ND | 5.41 | ND | ND | ND | ND | ND | ND |
| 121 | 2,4,4-trimethyl-Hexane | 1617 | MS | ND | ND | ND | ND | ND | 0.12 | ND | ND | ND | ND | ND | ND |
| 122 | 2,6-dimethyl-Undecane | 1617 | MS,RI | ND | ND | ND | ND | ND | 0.13 | ND | ND | ND | ND | ND | ND |
| 123 | 3,3-dimethyl-Hexane | 1615 | MS | ND | ND | ND | ND | ND | ND | ND | 0.13 | ND | ND | ND | ND |
| 124 | 1-(2-propenyloxy)-Butane | 707 | MS | ND | ND | ND | ND | ND | ND | 4.69 | ND | 2.71 | ND | 0.18 | ND |
| 125 | 6-ethyl-2-methyl-Decane | 1400 | MS | ND | ND | ND | ND | ND | ND | 0.26 | ND | ND | ND | ND | ND |
| 126 | 5-ethyl-Undecane | 1614 | MS | ND | ND | ND | ND | ND | ND | 0.20 | ND | ND | ND | ND | ND |
| 127 | 1,1-dimethyl-Cyclopentane | 933 | MS | ND | ND | ND | ND | 0.35 | ND | ND | ND | ND | ND | ND | ND |
| 128 | Azetidine | 966 | MS | ND | ND | ND | ND | 0.28 | ND | ND | ND | ND | ND | ND | ND |
| 129 | Octane | 706 | MS,RI | ND | ND | ND | ND | ND | ND | ND | ND | 0.33 | ND | ND | ND |
|  | Total |  |  | 0.53 | 1.65 | 9.71 | 0.28 | 0.63 | 9.42 | 5.35 | 0.13 | 5.83 | 0.13 | 0.18 | 0.58 |
|  | **Benzenes** |  |  |  |  |  |  |  |  |  |  |  |  |  |  |
| 130 | Styrene | 884 | MS,RI | ND | ND | ND | ND | ND | 2.08 | 2.74 | 3.05 | 2.87 | 0.41 | 0.53 | 0.36 |
| 131 | Benzyl nitrile | 1146 | MS | ND | ND | ND | ND | ND | 3.48 | ND | ND | ND | ND | ND | ND |
| 132 | 1-isocyano-3-methyl-Benzene | 1147 | MS | ND | ND | ND | ND | ND | 4.52 | ND | 3.23 | 1.86 | ND | ND | ND |
| 133 | (1-pentylhexyl)-Benzene | 1442 | MS | ND | ND | ND | ND | ND | 0.48 | ND | ND | ND | ND | ND | ND |
| 134 | 2-methyl-Benzonitrile | 1143 | MS | ND | ND | ND | ND | ND | ND | 4.72 | ND | ND | ND | ND | ND |
| 135 | 1-isocyano-4-methyl-Benzene | 1129 | MS | ND | ND | ND | ND | ND | ND | ND | ND | 1.65 | ND | ND | 3.82 |
| 136 | m-Aminophenylacetylene | 1131 | MS | ND | ND | ND | ND | ND | ND | ND | ND | ND | ND | 1.64 | ND |
|  | Total |  |  | ND | ND | ND | ND | ND | 10.57 | 7.46 | 6.28 | 6.38 | 0.41 | 2.17 | 4.19 |
|  | **Ethers** |  |  |  |  |  |  |  |  |  |  |  |  |  |  |
| 137 | Dimethyl ether | 683 | MS,RI | 4.51 | 10.73 | 6.43 | 3.82 | ND | 9.52 | 9.75 | 14.91 | 6.15 | ND | ND | ND |
| 138 | Disulfide, dimethyl | 739 | MS,RI | ND | ND | ND | ND | 1.87 | 17.99 | 8.22 | 4.79 | 1.70 | 3.42 | 6.36 | 8.05 |
| 139 | Ethoxy-ethene | 687 | MS | ND | ND | ND | ND | ND | ND | ND | 2.62 | ND | ND | ND | ND |
|  | Total |  |  | 4.51 | 10.73 | 6.43 | 3.82 | 1.87 | 27.51 | 17.98 | 22.32 | 7.85 | 3.42 | 6.36 | 8.05 |
|  | **Azoles** |  |  | ND | ND | ND | ND | ND | ND | ND | ND | ND | ND | ND | ND |
| 140 | 3,5-Diamino-1,2,4-triazole | 1068 | MS | ND | 0.25 | ND | ND | ND | ND | ND | ND | ND | ND | ND | ND |
| 141 | 1H-Pyrazole-4-carbonitrile | 821 | MS | ND | ND | ND | ND | ND | 0.66 | ND | ND | ND | ND | ND | ND |
| 142 | 3-amine, 4-methyl-4H-1,2,4-Triazol | 1043 | MS | ND | ND | ND | ND | ND | 0.15 | ND | ND | ND | ND | ND | ND |
| 143 | Benzofuroxan | 1275 | MS | ND | ND | ND | ND | ND | ND | ND | ND | ND | ND | ND | 0.07 |
| 144 | 2,5-dimethyl-2H-Tetrazole | 880 | MS | ND | ND | ND | ND | 0.58 | ND | ND | ND | ND | ND | ND | ND |
| 145 | 1H-1,2,4-Triazole | 941 | MS | ND | ND | ND | ND | ND | ND | ND | ND | 0.48 | ND | ND | ND |
|  | Total |  |  | 0.00 | 0.25 | 0.00 | 0.00 | 0.58 | 0.82 | ND | ND | 0.48 | ND | ND | 0.07 |
|  | **Olefins** |  |  |  |  |  |  |  |  |  |  |  |  |  |  |
| 146 | Spiro[2,4]hepta-4,6-diene | 748 | MS | ND | ND | 0.25 | ND | ND | ND | 1.57 | 7.42 | ND | ND | 2.33 | ND |
| 147 | 3-Ethyl-4-methyl-2-pentene | 865 | MS | ND | ND | ND | ND | ND | 0.19 | 0.59 | 0.29 | ND | 0.11 | 0.22 | 0.22 |
| 148 | 3-propoxy-1-Propene | 706 | MS | ND | 3.22 | ND | ND | 1.22 | ND | ND | ND | ND | ND | ND | ND |
| 149 | Cyclopropene | 859 | MS | ND | ND | ND | ND | ND | ND | ND | ND | ND | 2.39 | ND | ND |
| 150 | Fumaronitrile | 921 | MS | ND | ND | ND | ND | ND | ND | ND | ND | ND | ND | 1.20 | ND |
| 151 | 1-ethyl-Cyclohexene | 878 | MS | ND | ND | ND | ND | ND | ND | ND | ND | 0.40 | ND | ND | ND |
| 152 | 3-ethyl-2-methyl-1,3-Hexadiene | 1002 | MS | ND | ND | ND | ND | ND | ND | ND | ND | 1.82 | ND | ND | ND |
|  | Total |  |  | ND | 3.22 | 0.25 | ND | 1.22 | 0.19 | 2.16 | 7.71 | 2.22 | 2.50 | 3.75 | 0.22 |
|  | **Others** |  |  |  |  |  |  |  |  |  |  |  |  |  |  |
| 153 | 2,5-Dimethylpyrimidine | 907 | MS | ND | ND | ND | 3.41 | ND | 3.96 | ND | ND | 2.59 | 3.81 | ND | ND |
| 154 | 1-ethyl-2,3-dihydro-1H-Indene | 1282 | MS | 0.40 | ND | ND | ND | ND | ND | ND | 1.78 | ND | ND | ND | ND |
| 155 | Glycidol | 709 | MS | ND | ND | ND | ND | ND | 2.85 | ND | ND | ND | ND | ND | ND |
| 156 | Dimethyl trisulfide | 964 | MS,RI | ND | ND | ND | ND | 0.13 | 4.96 | 6.53 | 5.96 | 4.47 | 1.88 | 2.18 | 10.09 |
| 157 | Aminoacetonitrile | 1087 | MS | ND | ND | ND | ND | ND | 0.26 | ND | ND | ND | ND | ND | ND |
| 158 | Azulene | 1188 | MS | ND | ND | ND | ND | ND | 1.65 | ND | ND | ND | ND | ND | ND |
| 159 | N-Dimethylaminomethyl-tert.-butyl-isopropylphosphine | 1194 | MS | ND | ND | ND | ND | ND | ND | ND | 0.21 | ND | ND | ND | ND |
| 160 | 1,2,3,4-tetrahydro-quinoline | 1264 | MS | ND | ND | ND | ND | ND | ND | 0.94 | ND | ND | ND | ND | ND |
| 161 | Propanedinitrile | 1058 | MS | ND | ND | ND | ND | ND | ND | ND | ND | ND | 2.08 | ND | ND |
| 162 | Cyanogen | 1134 | MS | ND | ND | ND | ND | ND | ND | ND | ND | ND | 1.10 | ND | ND |
| 163 | Propyne | 736 | MS | ND | ND | ND | ND | ND | ND | ND | ND | ND | ND | 3.35 | 0.37 |
|  | Total |  |  | 0.40 | ND | ND | 3.41 | 0.13 | 13.68 | 7.48 | 7.95 | 7.06 | 8.86 | 5.53 | 10.46 |

*^*^*NE represents sesame meal without enzyme treatment; CP, HP, and PP represent sesame meal enzymatically treated by cellulase-papain complex, hemicellulase-papain complex, and pectinase-papain complex, respectively. Numbers 120, 150, 180 correspond to the sesame meal roasted at 120°C, 150°C, and 180°C, respectively.

**Table S3** The particle size distribution of the sesame paste.

| Sample^*^ | Particle size distribution (μm)^**^ | | |
| --- | --- | --- | --- |
|  | D10 | D50 | D90 |
| NEP120 | 16.8±0.2^a^ | 144.5±3.1^a^ | 300.7±4.7^a^ |
| CPP120 | 9.7±0.1^e^ | 76.2±1.4^e^ | 207.4±3.3^e^ |
| HPP120 | 10.6±0.2^c^ | 82.6±2.0^d^ | 234.9±3.4^c^ |
| PPP120 | 10.2±0.2^d^ | 97.9±2.2^c^ | 240.2±3.5^b^ |
| NEP150 | 12.6±0.1^b^ | 138.4±2.8^b^ | 301.0±4.7^a^ |
| CPP150 | 9.2±0.1^f^ | 59.3±1.6^h^ | 143.3±2.9^i^ |
| HPP150 | 8.4±0.1^g^ | 72.0±1.9^f^ | 176.1±3.1^f^ |
| PPP150 | 8.4±0.1^g^ | 68.6±1.7^g^ | 205.5±3.4^e^ |
| NEP180 | 7.6±0.1^h^ | 58.6±1.5^h^ | 221.5±3.8^d^ |
| CPP180 | 7.2±0.1^i^ | 40.7±1.2^j^ | 147.4±2.6^i^ |
| HPP180 | 4.8±0.1^j^ | 52.6±1.4^i^ | 153.9±3.4^h^ |
| PPP180 | 7.1±0.1^i^ | 56.2±1.6^h^ | 164.8±3.2^g^ |

^*^NEP represents the paste made from the sesame meal without enzyme treatment; CPP, HPP, and PPP represent the paste made from the sesame meal enzymatically treated by cellulase-papain complex, hemicellulase-papain complex, and pectinase-papain complex, respectively. Numbers 120, 150, 180 correspond to the sesame meal roasted at 120°C, 150°C, and 180°C, respectively.

^**^D90 (μm) indicates the particle size corresponding to a particle size distribution of 90%; D50 (μm) indicates the average particle size of paste; D10 (μm) indicates the particle size corresponding to a particle size distribution of 10%. ^a–j^ superscript indicates significant (P < 0.05) differences among the different ball milling times.

**Table S4** Rheological parameters of the Ostwald de Waele model (*τ* = *K*$\dot{\gamma}$*^n^*) of the five paste samples.

| Sample^*^ | K (Pa⋅s^n^)^**^ | n | R^2^ |
| --- | --- | --- | --- |
| NEP120 | 2.52±0.09 | 0.71±0.01 | 0.997 |
| CPP120 | 1.53±0.04 | 0.83±0.01 | 0.998 |
| HPP120 | 1.50±0.03 | 0.84±0.01 | 0.999 |
| PPP120 | 1.52±0.08 | 0.86±0.02 | 0.986 |
| NEP150 | 3.57±0.10 | 0.68±0.01 | 0.998 |
| CPP150 | 2.17±0.04 | 0.72±0.01 | 0.999 |
| HPP150 | 1.90±0.06 | 0.74±0.02 | 0.997 |
| PPP150 | 1.62±0.04 | 0.79±0.01 | 0.998 |
| NEP180 | 4.65±0.08 | 0.64±0.02 | 0.999 |
| CPP180 | 2.44±0.04 | 0.71±0.01 | 0.999 |
| HPP180 | 1.82±0.03 | 0.72±0.01 | 0.999 |
| PPP180 | 1.55±0.02 | 0.74±0.01 | 0.999 |

^*^NEP represents the paste made from the sesame meal without enzyme treatment; CPP, HPP, and PPP represent the paste made from the sesame meal enzymatically treated by cellulase-papain complex, hemicellulase-papain complex, and pectinase-papain complex, respectively. Numbers 120, 150, 180 correspond to the sesame meal roasted at 120°C, 150°C, and 180°C, respectively.

^**^*K* indicates consistency coefficients; *n* indicates flow behavior indices; *R*^2^ indicates coefficients of determination; Data are means ± SD.

**Fig. S1.** Hot-air diagram of volatile compounds found in twelve sesame meal samples.

NE represents sesame meal without enzyme treatment; CP, HP, and PP represent sesame meal treated with cellulase-papain complex, hemicellulase-papain complex, and pectinase-papain complex, respectively. Numbers 120, 150, 180 correspond to the sesame meal roasted at 120°C, 150°C, and 180°C, respectively.

**NEP**

**CPP**

**HPP**

**PPP**

**120°C**

**150°C**

**180°C**

**50μm**

**50μm**

**50μm**

**50μm**

**50μm**

**50μm**

**50μm**

**50μm**

**50μm**

**50μm**

**50μm**

**50μm**

**Fig. S2.** Microstructure of the twelve sesame paste samples.

Nile red-stained lipids appear red; FITC-stained protein appears green in this image.

Norharman and Harman reference standards:

NE120: CP120:

HP120: PP120:

NE150: CP150:

HP150: PP150:

NE180: CP180:

HP180: PP180:

**Fig. S3.** Liquid chromatographic profiles of norharman and harman in all samples.

NE represents sesame meal without enzyme treatment; CP, HP, and PP represent sesame meal treated with cellulase-papain complex, hemicellulase-papain complex, and pectinase-papain complex, respectively. Numbers 120, 150, 180 correspond to the sesame meal roasted at 120°C, 150°C, and 180°C, respective.

NE120: CP120:

HP120: PP120:

NE150: CP150:

HP150: PP150:

NE180: CP180:

HP180: PP180:

**Fig. S4.** Chromatogram and mass spectrum of all samples.

NE represents sesame meal without enzyme treatment; CP, HP, and PP represent sesame meal treated with cellulase-papain complex, hemicellulase-papain complex, and pectinase-papain complex, respectively. Numbers 120, 150, 180 correspond to the sesame meal roasted at 120°C, 150°C, and 180°C, respective.
